# Filtering property of myelinated internode can change neural information representability and might trigger a compensatory action during demyelination

**DOI:** 10.1038/s41598-023-49208-9

**Published:** 2023-12-14

**Authors:** Sarbani Das, Koushik Maharatna

**Affiliations:** School of Electronics and Computer Science, University of Southampton, University Road, Southampton, SO17 1BJ UK

**Keywords:** Computational biology and bioinformatics, Neuroscience, Physiology

## Abstract

In this paper, for the first time, we showed that an Internode Segment (INS) of a myelinated axon acts as a lowpass filter, and its filter characteristics depend on the number of myelin turns. Consequently, we showed how the representability of a neural signal could be altered with myelin loss in pathological conditions involving demyelinating diseases. Contrary to the traditionally held viewpoint that myelin geometry of an INS is optimised for maximising Conduction Velocity (CV) of Action Potential (AP), our theory provides an alternative viewpoint that myelin geometry of an INS is optimised for maximizing representability of the stimuli a fibre is meant to carry. Subsequently, we show that this new viewpoint could explain hitherto unexplained experimentally observed phenomena such as, shortening of INS length during demyelination and remyelination, and non-uniform distribution of INS in the central nervous system fibres and associated changes in diameter of nodes of ranvier along an axon. Finally, our theory indicates that a compensatory action could take place during demyelination up to a certain number of loss of myelin turns to preserve the neural signal representability by simultaneous linear scaling of the length of an INS and the inner radius of the fibre.

## Introduction

Propagation characteristics of an AP train in myelinated axons is highly dependent on the geometry of an INS. But how the geometric parameters of an INS—length ($L$), inner radius ($r$) and number of myelin turns ($M$)—are related to each other and what exact criteria govern their choice is not yet clearly understood. Traditionally accepted viewpoint, first proposed by Rushton^1^ is that an INS geometry is optimized to maximize CV of an AP. Based on this assumption, his foundational theoretical work^1^ showed that an INS geometry could be specified by an optimal $g$-ratio (the ratio of the inner diameter of the fibre $({d}_{in})$ to its outer diameter $(D)$) and a $L/D$ ratio. This theory was later questioned by several studies as follows:

1. The $g$-ratio calculated in^1^ was $\sim 0.6$ in contrast to experimentally found $\sim 0.7-0.8$.
2. In the context of CNS fibres, it was pointed out that CV maximisation could not be the only criterion for INS geometry optimisation^2,3^.
3. Correlation of $L$ and $D$ didn’t hold true since under pathological conditions involving demyelination, the INS length becomes significantly shorter and upon remyelination still remains abnormally short despite more complete recovery of fibre diameter and myelin thickness^4–9^.
4. Experiments and simulations also found that an INS length and the diameter of the subsequent Nodes of Ranvier (NR) could be highly variable along the length of a CNS axon^10,11^.
5. Myelin geometry is highly plastic, and the thickness of its sheath may change dynamically depending upon activity of a neuron^12–14^.

Despite these apparent contradictions, Rushton’s central idea that INS geometrical parameters must be chosen for maximizing CV prevailed until now and CV measurement has become the cornerstone for estimating AP slow-down due to pathological conditions involving demyelination. But, if the cable theory model describing an INS as a Resistance–Capacitance (RC) circuit is correct, then CV must be a function of the input signal frequency, i.e., the AP firing rate. Since the main purpose of a nerve fibre is to carry a time-varying stimulus information encoded in the firing rate of an AP train^15,16^, which can vary widely depending on the stimulus intensity^17^, the CV must be different for different rates of AP firing representing different states of a stimulus. This raises the question of which CV value should be considered for the INS geometry selection.

On the other hand, it is imperative that an INS must support all the possible firing rates an AP train may assume to properly represent temporal variation of the stimuli a fibre is meant to carry—its Neural Code Capacity (NCC), with addition of some tolerance against possible malfunctions due to pathological process. Therefore, it seems logical to assume that an INS geometry is chosen to satisfy a fibre’s required NCC. The following system-level argument supports such an assumption:

Let’s consider an INS as a Single Input Single Output (SISO) system between the *i*th and ${(i+1)}$th NR. Since it behaves like a RC circuit, its frequency-dependent input–output relation could be given by $G(f)={v}_{out}(f)/{v}_{in}(f)$ where, $G(f)$ is the system’s gain, and ${v}_{out}(f)$ and ${v}_{in}(f)$ are its frequency-dependent output and input voltage (input to ${(i+1)}$th NR and output of *i*th NR) respectively. If the goal is undisrupted AP propagation, then the condition ${v}_{out}(f)\ge {v}_{TH}$, where ${v}_{TH}$ is the threshold voltage of AP firing at the ${(i+1)}$th NR, must hold true for the entire AP train frequency range necessary to represent the possible temporal variations of a stimulus. In addition, the above-mentioned condition must also hold true for the range of frequency components contained in an AP waveform itself so that the AP shape and its peak amplitude is preserved. Therefore, $G(f)$ must be chosen in such a way that both these conditions are simultaneously satisfied. This means that selection of the resistances and capacitances and hence, the three geometrical parameters of an INS—$r$, $M$ and $L$ must be chosen according to the required $G(f)$.

Using this concept and the Double Cable (DC) model of INS^18^, in this paper, we first prove that an INS is a low-pass filter and its NCC is a linear function of combination of $r$, $M$ and $L$, and irrespective of PNS or CNS; $r$, $M$ and $L$ are linearly interdependent. This leads to a mathematical equation describing how changes in $M$ affect representability of a stimulus and thereby altering the neural information. Subsequently, following the viewpoint that the INS geometrical parameters are chosen for satisfying the requirement of a certain NCC, we show that the experimental observation of shortening of $L$ during demyelination is an outcome of a compensatory mechanism where $r$ and $L$ simultaneously scale linearly with $M$ to preserve the original NCC of a fibre. In this context we prove that although one may observe reduction of CV during myelin loss, the Conduction Delay (CD) can still remain constant.

Our theory is based on simulation experiments with previously published data for 6 PNS and 2 CNS fibres and applying well-established system analysis techniques while considering an isolated INS as a SISO system. For ease of discussion, we denote the maximum possible AP train frequency at the input of an INS for which the condition ${v}_{out}(f)\ge {v}_{TH}$ holds true, i.e., the NCC of a fibre, as ${f}_{L}$. We also consider that the upper limit of biologically relevant range of ${f}_{L}$ is 1 KHz considering that the observed (so far) absolute refractory period of an AP is $\sim 1ms$ and therefore is the minimum time required to generate two successive APs.

## Results

### INS behaves as a low-pass filter with ${f}_{L}$ higher than biologically relevant range in normal operating condition

**Figure 1:**
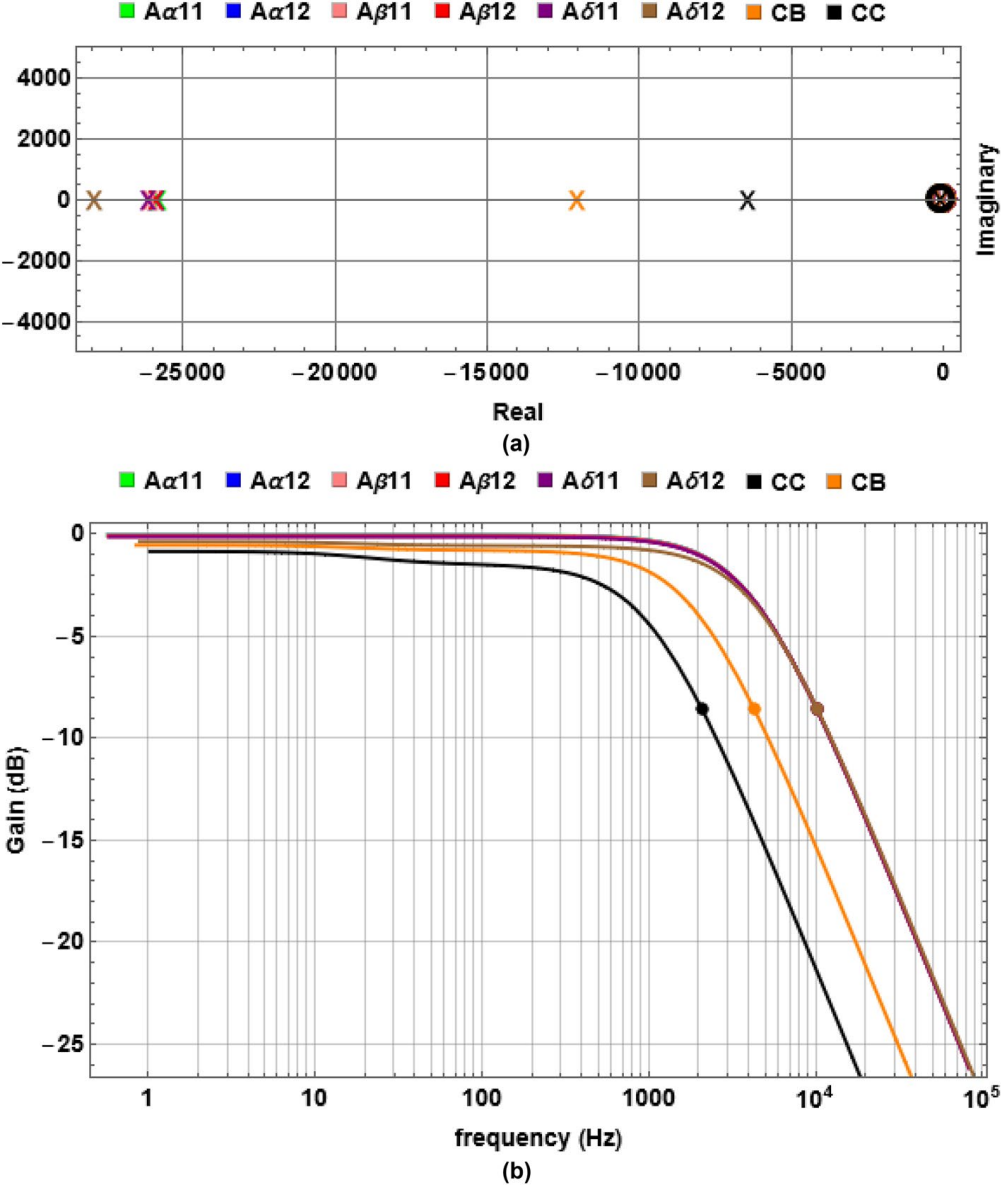
The frequency characteristics of the INS for the fibres under consideration (see Section “Geometrical parameters” in Section “Materials and methods” for explanation of the legends and Table 2 for their dimensions). (**a**) shows the pole-zero plots with the circles representing zeros and “ × ” representing the poles. Note the coincident poles and zeros for all the fibres close to the origin. (**b**) The frequency dependent gain characteristics of all the fibres. Filled circles denote the ${f}_{L}$ for each fibre calculated following Eq. (13), see Section “Materials and methods” Section “INS system modelling and f_L_ calculation”. All the PNS fibres exhibit identical characteristics and their ${f}_{L}$ is coincident while the CNS fibres show their individual characteristics.

Equation (12) (see Section “Materials and methods”) implies that an INS is a 2nd order system, and its frequency response should be of bandpass type, i.e., it will pass a certain frequency range unattenuated while attenuating the other frequencies that do not fall within that band. A straightforward way to determine such a passband is to examine the pole-zero plot of the system Transfer Function (TF; Section “Materials and methods”) for each of the fibres as shown in Fig. 1a. As expected, each TF results in two poles and one zero in accordance with a bandpass system’s characteristic. But, surprisingly, for all the fibres, their only zeros coincide with one of their two poles compensating each other’s effect leaving only the other pole to dominate the system’s behaviour. This effectively makes an INS a lowpass filter. More interestingly, the dominant poles of all the PNS fibres are located very close to each other implying that despite having different values of $L$, $r$ and $M$ (Table 2, Section “Materials and methods”), all the PNS fibres would exhibit similar time and frequency response characteristics. On the other hand, the dominant poles of the CNS fibres are separated significantly to show that each of them may exhibit their own characteristic time and frequency response property. To verify this and to find the ${f}_{L}$ (following the process outlined in Section “Materials and methods”) for each fibre we derived the Bode Magnitude plot from each fibre’s TF as shown in Fig. 1b. Consistent with Fig. 1a, all the fibres show lowpass filter characteristics with all the PNS fibres exhibiting similar frequency-dependent gain characteristics with their ${f}_{L}$ coincided. Overall, this is surprising since the individual PNS fibres have different INS geometries (see Table 2), and thus are expected to show different gain characteristics. The only possible scenario under which such behaviour can occur is if $L$, $r$ and $M$ are interrelated so that multiple but appropriate combinations of them can lead to the same ${f}_{L}$. It is worth noting that the numerical values of $g$ and $\gamma$ (see Table 2) for all the PNS fibres are the same despite having different geometries and therefore strongly suggests such interdependency.

On the other hand, the CNS fibres show their individual characteristics with lower gain and ${f}_{L}$ compared to the PNS fibres. Interestingly, although their $g$-values are close to those of the PNS fibres, their $\gamma$-values differ significantly not only compared to the PNS fibres but also among themselves, which could be suggestive to their location-specific unique characteristics. However, such a different behaviour precludes a definitive conclusion that $L$, $r$ and $M$ are interrelated. But, in all the cases, ${f}_{L }>1$ KHz implies that no AP disruption could take place within an INS in their normal operating condition.

### Representability of temporal variation of a stimulus could change depending upon the myelination state of a fibre

**Figure 2:**
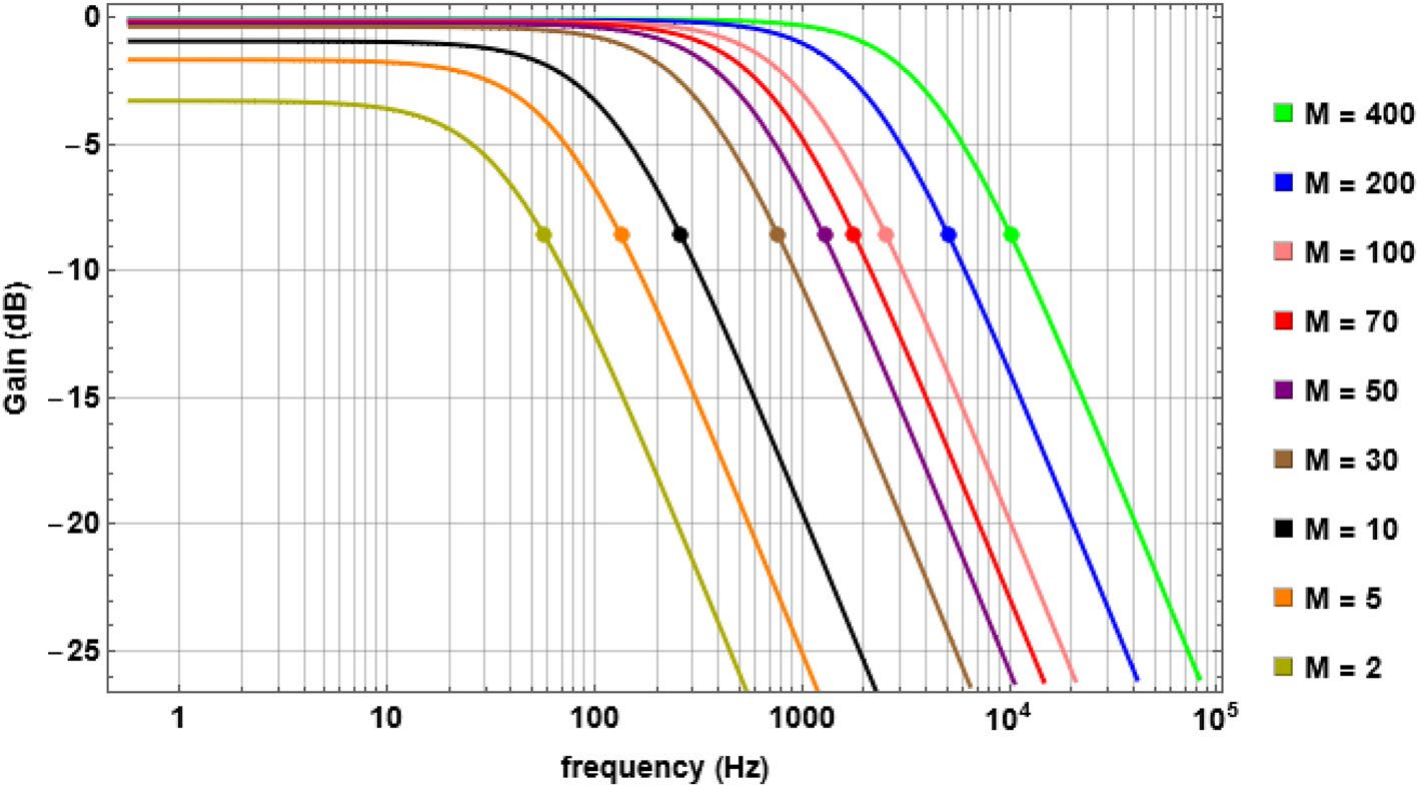
The variation of frequency-dependent gain characteristics of $A\alpha 11$ fibre with changes of myelin turns ($M$). Filled circles denote ${f}_{L}$ in each case—note its shift toward the higher frequency range with increase in $M$ and vice-versa.

Next, we progressively changed $M$ for each fibre while keeping their $L$ and $r$ constant and extracted ${f}_{L}$, (Section “Materials and methods). The results are shown in Fig. 2 for the case of A $\alpha 11$ fibre (see Section “Materials and methods” for its definition) as an illustrative example (for the corresponding figures for all the other fibres see Fig. S1 in SI). As $M$ decreases, ${f}_{L}$ moves toward the lower frequency band—reducing the fibre’s NCC—eventually moving below $1$ KHz, in this case, when $M<50$.

The implication could be explained by taking the specific example of ${f}_{L}=767.8$ Hz when $M=30$. At this condition only those states of a stimulus that could be represented by AP firing rate $\le 767.8$ Hz will pass through the INS while any state representation requiring AP firing frequency above that will be blocked within the INS—effectively filtering out part of the original neural code. An illustrative example has been shown in Fig. S2 (SI) with AP train of 1 KHz frequency. We observed the same pattern for all the fibres: ${f}_{L}$ changes linearly with $M$ (see Fig. S3, SI) implying that to reach a certain NCC, a fibre needs a certain minimum $M$. But the rate of change of ${f}_{L}$ with respect to $M$ appears to be steeper for the fibres with smaller $r$ and $L$. This suggests that the locus of ${f}_{L}$ might not only be dependent on $M$ but possibly on $r$ and $L$ too. Therefore, it is more prudent to study the behavior of ${f}_{L}$ as a combined effect of these three parameters.

At the same time, we found that the reduction of $M$ will not affect the shape and amplitude of an AP since most of its spectral power is concentrated within a very narrow low-frequency band of 1–5 Hz with the maximum at $1.9$ Hz (see Fig. S4, SI). Therefore, the shape and amplitude of an AP could only be changed significantly for ${f}_{L}$ $\le 1.9$ Hz which is at least an order lower than the lowest value of ${f}_{L}$ corresponding to $M=1$.

### *L*, *r* and *M* are linearly interdependent irrespective of the type of nerve fibres

**Figure 3:**
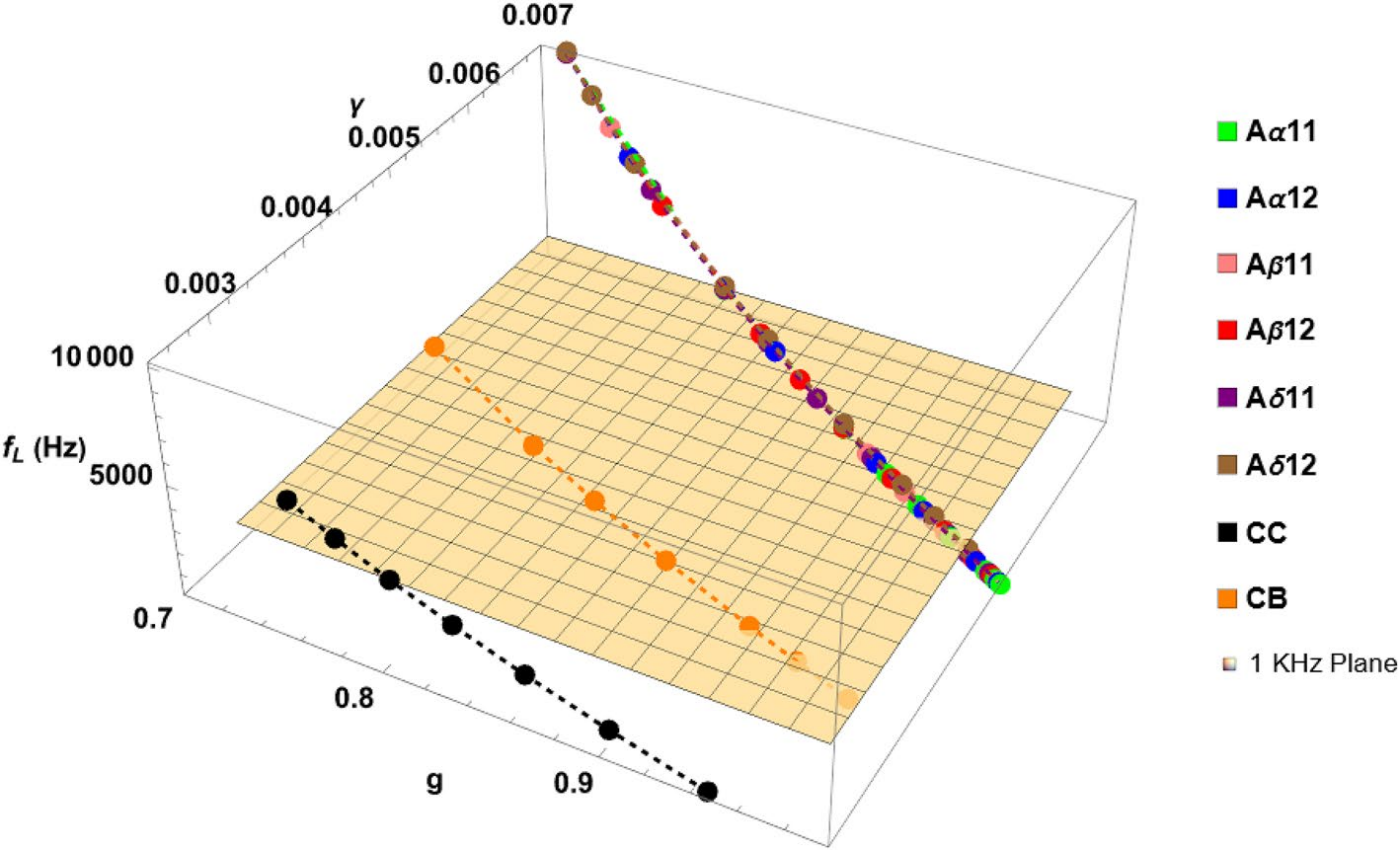
The loci of ${f}_{L}$ with respect to variation in $g$ and $\gamma$. All the PNS fibres show the same behaviour, and loci of their ${f}_{L}$ follow a linear curve. On the contrary, the CNS fibres show individual behaviour although still exhibit linear relation with $g$ and $\gamma$ given by Eq. (1).

To study how ${f}_{L}$ varies as a combined effect of $r$, $L$ and $M$, we plotted ${f}_{L}$ for each $M$ for all the fibres against their corresponding $g$ and $\gamma$ as shown in Fig. 3. Surprisingly, instead of being scattered around on a g-$\gamma$ surface, the loci of ${f}_{L}$ for all the PNS fibres overlap with each other forming a single curve as shown by the brown dashed trajectory in Fig. 3, that could be represented by the fitted linear Eq. (1) (95% confidence bounds, R^2^ = 0.99):

1 $${f}_{L}=ag+b\gamma +c$$

where $a$ $= -1344.98$ $(-4101.07, 1411.11),$ $b = 4.847\times {10}^{6} (4.45\times {10}^{6} , 5.245\times {10}^{6} )$ and $c = -22857.5 (-27568.8, -18146.3)$. Although the loci of ${f}_{L}$ for the CNS fibres do not show such overlapping, they follow the same linear functional form, and the fitted model (95% confidence interval and adjusted R^2^ = 1 in both cases) could be given by the following parameters:

**Table:** 

Fibre name	$a$	$b$	$c$
CC	$-29.437$ $\left(-53.655,-5.216\right))$	$2.177\times {10}^{6}$ $(2.17\times {10}^{6},$ $2.184\times {10}^{6}$)	$-4781.343$ $(-4821.47, -4741.22)$
CB	$-17.976$ $(-41.54, 5.59)$	$3.346\times {10}^{6}$ $(3.3413\times {10}^{6}, 3.351 \times {10}^{6}$)	$-11186.2$ $(-11226.4, -11146.1)$

This means, irrespective of fibre types, ${f}_{L}$ is a linear function of $g$ and $\gamma$, and every possible ${f}_{L}$ of all the PNS fibres could be specified by a unique tuple $(g, \gamma )$ whilst in the case of CNS fibres the same is true but in fibre-specific manner. Therefore, for a specific ${f}_{L}$ the following two conditions hold:

2a $$g\gamma ={C}_{1}$$

2b $$\frac{g}{\gamma }= {C}_{2}$$

where ${C}_{1}$ and ${C}_{2}$ are two unique constants specific to a ${f}_{L}$ for a fiber. Substituting Eqs. (7k) and (7l) (Section “Materials and methods”) in Eqs. (2a) and (2b) we get

3a $$r={C}_{1}L$$

3b $$\frac{rL}{{(r+2{t}_{m}M)}^{2}}={C}_{2}$$

Substituting $r$ from Eq. (3a) into Eq. (3b) and solving for $L$ we get

4 $$L=-\frac{2\sqrt{{\mathrm{C}}_{2}}M{t}_{m}}{-\sqrt{{\mathrm{C}}_{1}}+{\mathrm{C}}_{1}\sqrt{{\mathrm{C}}_{2}}}, L=-\frac{2\sqrt{{\mathrm{C}}_{2}}M{t}_{m}}{\sqrt{{\mathrm{C}}_{1}}+{\mathrm{C}}_{1}\sqrt{{\mathrm{C}}_{2}}}$$

Since all the parameters on the right-hand side of both equations are positive, the second solution does not exist since $L$ cannot be negative, leaving only the first solution. For that solution to exist the following condition must be satisfied

5 $$\sqrt{{C}_{1}}>{C}_{1}\sqrt{{C}_{2}};\mathrm{ i}.\mathrm{e}.,\sqrt{{C}_{1}{C}_{2}}<1$$

Since both $g$ and $\gamma$ are always $<1$ and ${C}_{2}\propto 1/{M}^{2}$, Eq. (5) is always satisfied for $M>0$ which means that $L$ and $M$ are linearly proportional. Combining this with Eq. (3a), proves that $r, L$ and $M$ are linearly dependent on each other, and this is valid both for a PNS and CNS fibre. That means, depending on a ‘desired’ NCC, irrespective of the type of a fibre, $r, L$ and $M$ are chosen to define the geometric structure of an INS following a linear interdependency rule, that allows unperturbed AP generation at the next NR for all operating frequencies up to ${f}_{L}$, which defines the upper limit of the NCC.

A consequence of Eq. (4) is, to ensure the full range representability of temporal variation of a stimulus, i.e., ${f}_{L}\ge 1$ KHz, there exists a limiting $L/M$ value for each fibre which could be calculated as follows:

Let $({g}_{1}, {\gamma }_{1})$ be the tuple at which ${f}_{L}$ = 1 kHz for a fibre and the corresponding constants in Eqs. (3a) and (3b) are $({C}_{11}, {C}_{12})$. Therefore, from Eq. (4) we get,

6 $$\frac{L}{M}=-\frac{2\sqrt{{\mathrm{C}}_{12}}{t}_{m}}{-\sqrt{{\mathrm{C}}_{11}}+{\mathrm{C}}_{11}\sqrt{{\mathrm{C}}_{12}}}$$

From Fig. 3, since all the PNS fibres intersect the ${f}_{L}$ = 1 KHz plane at a unique point $({g}_{1}=0.961, {\gamma }_{1}=0.0052)$, from Eq. (6) $L/M$ $\le 0.5 \mu$ m is the condition for a PNS fibre to keep its ${f}_{L} \ge 1$ KHz. Similar limiting conditions for the CNS fibres could also be derived but since they exhibit their individual characteristics, such limiting condition needs to be calculated in fibre-specific way.

### Loss of myelin turns under pathological conditions might trigger a compensatory mechanism to preserve a fibre’s NCC by dynamically scaling $r$ and $L$ linearly

**Figure 4:**
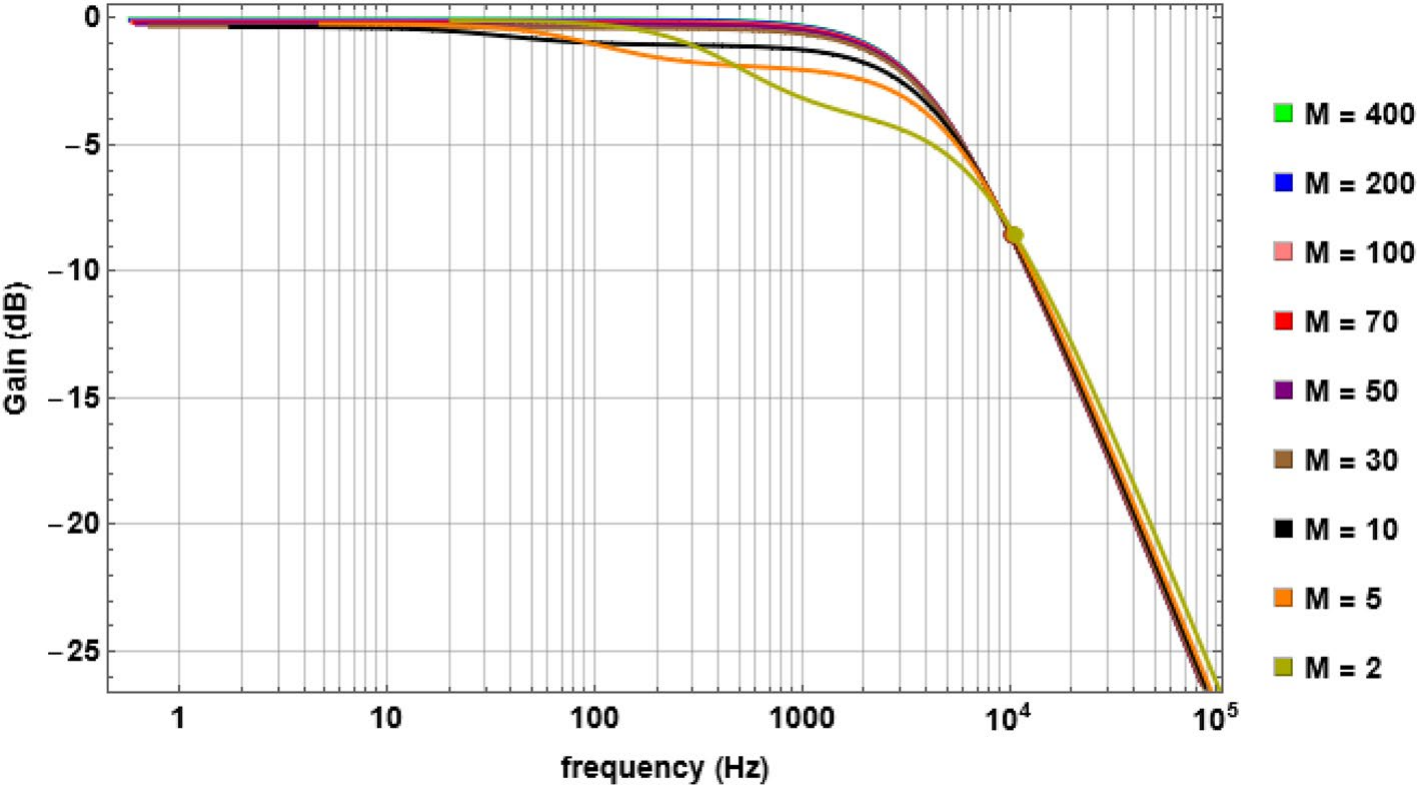
Gain characteristics of the $A\alpha 11$ fibre during compensation shows almost the same characteristics with ${f}_{L}$ coincident for all the curves. Solid circles show the ${f}_{L}$ for each TF calculated with $r$ and $L$ estimated using Eqs. (3a) and (4) for different values of $M$. Note that ${f}_{L}=10$ KHz is maintained even when $M$ reaches to 2.

Since both $r$ and $L$ are linearly dependent on $M$, with reduction of $M$ during pathological conditions related to demyelination, there must be different possible combinations of $r$ and $L$ for which a unique tuple $(g, \gamma )$ and hence a unique ${f}_{L}$ could remain constant if a fibre ‘intends’ to maintain that ${f}_{L}$. To illustrate the scenario, let’s assume that the $A\alpha 11$ fibre intends to preserve its normal operating ${f}_{L}=10$ KHz (approx.) during such a scenario. From the values of $(g, \gamma )$ in Table 2 corresponding to ${f}_{L}=10$ KHz, using Eqs. (2a) and (2b), we calculate ${C}_{1}=0.005$ and ${C}_{2}=102.041$. Applying these values in Eqs. (4) and (3a), we calculated the values of $L$ and $r$ respectively for each value of $M$ from $400$ to $1$ (Fig. S5, SI). Subsequently, we derived the TFs of the INS for each of these $(r, L, M)$ combinations and plotted the corresponding Bode Magnitude plot in Fig. 4 which shows that even in this case the ${f}_{L}$ remains constant. We have observed the same behavior for all the fibres with different ${f}_{L}$ values. The experimental findings that during demyelination the INS length is shortened when interpreted based on the results from Fig. 4 clearly suggest an on-going compensatory mechanism to maintain a certain constant ${f}_{L}$, by simultaneous linear scaling of $r$ and $L$ with $M$ following Eqs. (3a) and (4). Although, as per our knowledge, no such direct experimental result is available with respect to $r$, our result suggests that the experimentally measured overall thinning of the outer diameter of a fibre in such a scenario must have the effect of reduction of $r$ embedded in it.

### CD remains constant during a compensatory scenario, but CV is reduced

**Figure 5:**
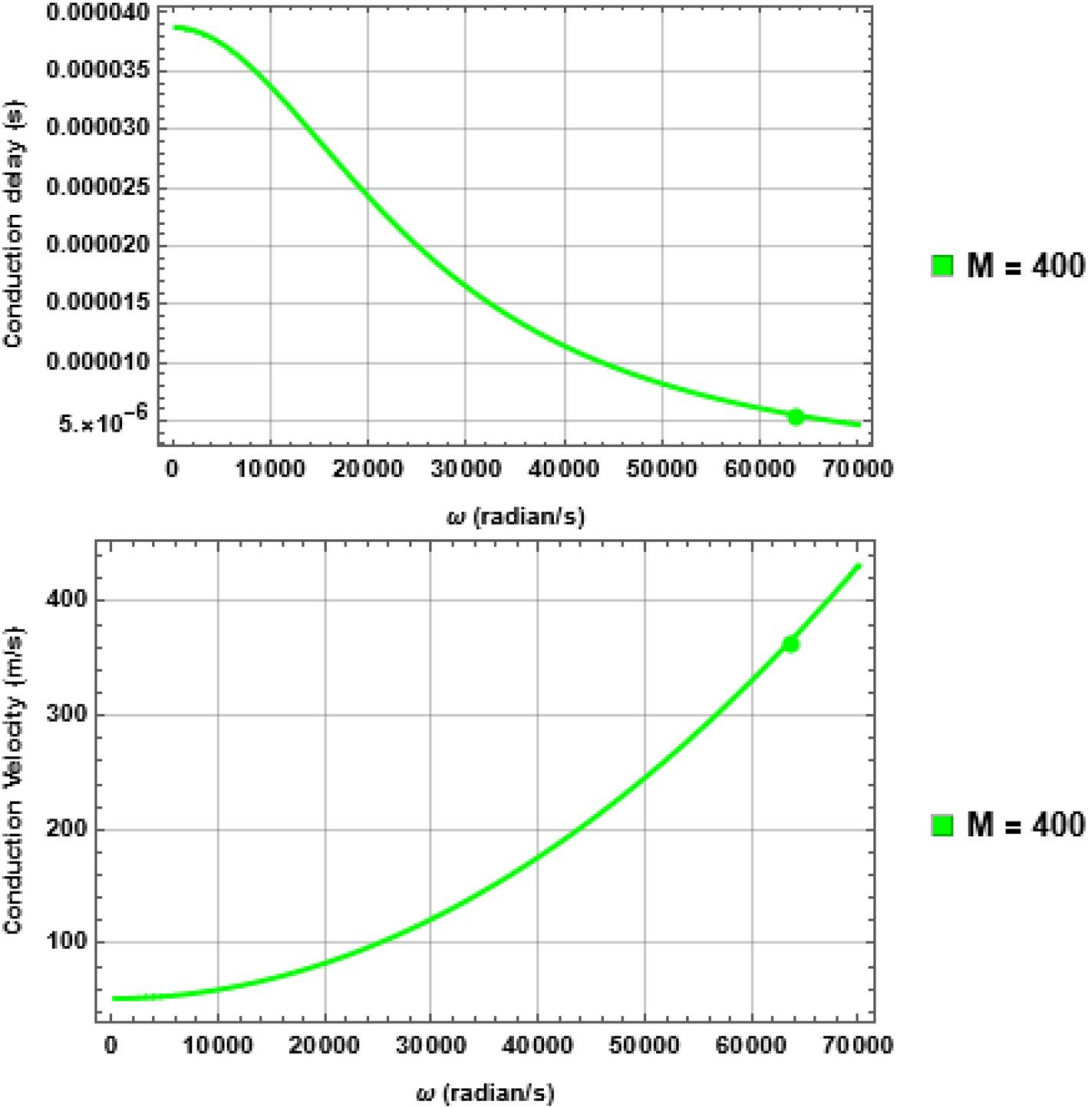
The frequency-dependent CD (top) and corresponding CV (bottom) for $A\alpha 11$ fibre in normal condition ($M=400$)—note that here we used angular frequency $\omega$ in the x-axis instead of linear frequency $f$ as the computation is done according to Eq. (14); Section “Materials and methods, which requires representing the phase angle in the unit of radians. The filled circles denote ${\omega }_{L}=2\pi {f}_{L}=62832\,rad/s$ corresponding to ${f}_{L}=10$ KHz. Note the value of CV below 1 kHz frequency ($\omega \le 6283\,rad/s)$ varies between 40 and 52 m/s—a value well within the experimentally found range since typically CV is measured within that frequency range.

Next, we calculate CV using the procedure described in Section “Materials and methods” by first calculating the CD. Figure 5 shows the result for $A\alpha 11$ fibre as an illustration with its corresponding geometrical parameters given in Table 2. Clearly, CD falls exponentially with increasing AP train frequency. Consequently, given a fixed $L$, the CV is also frequency dependent and increases exponentially with AP firing frequency.

**Figure 6:**
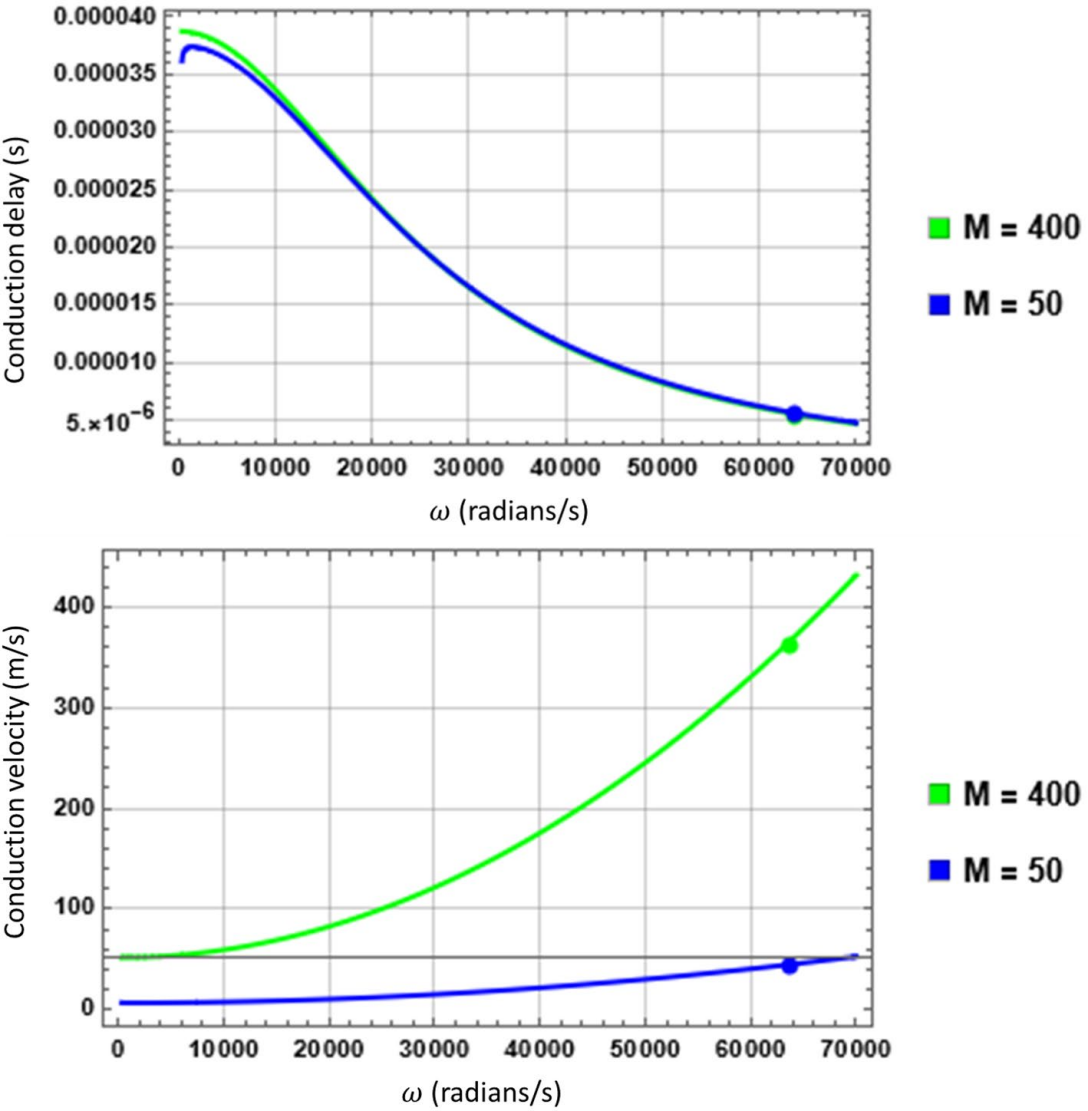
Comparison of CD and CV during compensation. Although the CD (Top) at ${f}_{L}=10$ KHz (${\omega }_{L}=62832 rad/s$) remains constant, in both cases of $M=400$ and $50$, the CV (Bottom) in the latter is much lower compared to the former. If measured within the range of 1 KHz ($\omega \le 6283 rad/s$), the CV will be even lower and consistent with experimentally measured range although this leads to a misinterpretation of the phenomenon.

But the situation is different during the compensatory scenario described in Section “Loss of myelin turns under pathological conditions might trigger a compensatory mechanism to preserve a fibre’s NCC by dynamically scaling *r* and *L* linearly”. Taking the same example of $A\alpha 11$ fibre, but this time with $M=50$ and its corresponding $L=0.25\,{\rm mm}$ (Fig. S5, SI), we illustrate the phenomenon in Fig. 6. Here it is evident that at the intended ${f}_{L}=10$ KHz, the CD remains constant at $5.371 \mu s$ for both $M=50$ and $400$ but the CV changed from $372 m/s$ to $44.68 m/s$ since in this case $L$ is reduced from $2\,{\rm mm}$ to $0.25\,{\rm mm}$ to keep CD constant. Therefore, it is not surprising that reduction of CV has been observed experimentally during loss of myelin but its interpretation that the AP signal slows down within the INS is not necessarily true—in fact it is suggestive to the ongoing compensatory action to preserve the NCC.

## Discussion

Our findings that there exist multiple combinations of $r, L$ and $M$ for preserving the unique tuple $(g, \gamma )$ and hence, a unique ${f}_{L}$ on one hand, support the general result derived in^1^ and is instrumental in qualitatively explaining several experimental observations that were in apparent contradiction with conclusions in^1^ as follows.

### In CNS the INS length is progressively reduced while the diameter of the NR increases progressively along the axon,^10,11,19^

Since our theory suggests that multiple combinations of $L, r$ and $M$ could satisfy the same ${f}_{L}$, it is not surprising to have different INS geometry along the length of an axon for preserving a certain ${f}_{L}$. As suggested in^19^, such distribution is possibly guided by the availability of local resources. To explain the progressive increase of NR diameter with shortening INS let’s assume that the typical length and radius of an INS and NR are (${L}_{INS}$, ${r}_{INS}$) and (${L}_{NR1}$, ${r}_{NR1}$) respectively. As ${L}_{INS}$ reduces, ${L}_{NR1}$ becomes proportionally longer to ${L}_{NR2}$ and ${r}_{NR1}$ changes to ${r}_{NR2}$ (say). Such a change in geometry will increase the delay in the NR from ${t}_{NR1}$ to ${t}_{NR2}$ while according to the findings in Section “CD remains constant during a compensatory scenario, but CV is reduced”, the delay introduced at INS (${t}_{INS}$) remains constant. Therefore, considering the INS-NR as a combined unit, the total delay changes from ${t}_{D1}={t}_{INS}+{t}_{NR1}$ to ${t}_{D2}={t}_{INS}+{t}_{NR2}$. Since NR is an unmyelinated section, the delay per unit length is similar to that of an unmyelinated fibre and thus, following the results of^1,20^, ${t}_{NR1} \propto {L}_{NR1}/\sqrt{{r}_{NR1}}$ and ${t}_{NR2} \propto {L}_{NR2}/\sqrt{{r}_{NR2}}$. If the final goal is to keep the NCC of the fibre constant, then the condition ${t}_{D1}={t}_{D2}$ must hold true which implies ${r}_{NR2}={r}_{NR1}{\left({L}_{NR2}/{L}_{NR1}\right)}^{2}$. Since $\left({L}_{NR2}/{L}_{NR1}\right)>1$, ${r}_{NR2}> {r}_{NR1}$ which qualitatively matches the experimental findings in^11^. On the other hand, if the target is to keep CV constant instead of the delay in NR, CV in an NR being proportional to $\sqrt{{r}_{NR}}$, we get ${r}_{NR2}={r}_{NR1}{\left({t}_{D1}/{t}_{D2}\right)}^{2}$. Since in this case $\left({t}_{D1}/{t}_{D2}\right)<1$, we get ${r}_{NR2}< {r}_{NR1}$ which is the opposite to the experimental observation in^11^.

### Shortening of INS during demyelination and remyelination

While our results directly explain why one might expect shortening of INS in the scenario of myelin loss ("Loss of myelin turns under pathological conditions might trigger a compensatory mechanism to preserve a fibre’s NCC by dynamically scaling *r* and *L* linearly"), this also gives a possible explanation to why the INS remains short during remyelination. In^13,14^, the authors found that the INS geometry changes with learning and might be a life-long process. This suggests that during remyelination one must not expect the regenerated myelin structure like a normal population immediately as the biological adjustments occur progressively over time influenced by learning-related plasticity property. Since our results show that multiple combinations of $L, r$ and $M$ are possible to attain a specific ${f}_{L}$, it is not hard to postulate that during remyelination the myelin geometry is configured only to support the ‘baseline’ NCC and over a longer observation window, one is likely to find more changes as suggested in^13,14^.

### CNS fibres might have unique properties depending upon their location

Experimental observations in^19,21^ showed that there exists regional difference in Oligodendrocyte properties in CNS. In our results described in Section "*L*, *r* and *M* are linearly interdependent irrespective of the type of nerve fibres", this is reflected by the similar functional form of ${f}_{L}$ for both the CNS fibres while differing by only the fitting constants which in essence encapsulate the values of the biological constants such as membrane resistivity, specific capacitance, and membrane thickness. Therefore, our theory is consistent with the above-mentioned experimental observations. On the other hand, the almost similar characteristics shown by the PNS fibres in this regard is possibly since each PNS fibre is myelinated by a single type of Schwann cell with similar property.

### Limit of compensatory action

The results presented in Section “Results” implies that in myelin loss-induced compensatory scenario it is possible to maintain the NCC by scaling down the INS geometrical parameters even when $M$ reaches to $1$. This is counterintuitive given the fact that partial myelin loss also can lead to pathological signal propagation conditions. Therefore, there must be a limit of compensation which the theory didn’t spell out explicitly. Numerical evaluation of Eqs. (3a) and (4) shows that given a certain ${f}_{L}$, to sustain such compensation, $r$ becomes $< 1 \mu m$ after a certain reduction of $M$ (Fig. S6, SI). It is a well-known fact that for the PNS fibres with such a small radius the advantage of myelination is lost due to energetics, speed, and mechanical stability requirements^1,20^. Therefore, this is an obvious point to stop any further compensation. On the other hand, any compensatory action will also elongate the adjacent NR and to mitigate the associated increase in delay, it’s diameter must be increased. This will bring the available space and resource requirements into question as described in^20^. These factors can certainly play a role in restricting the compensation action even earlier. However, appropriate directed experiments are needed to explore this area.

### Implications on neural information processing

The traditional view of neural information processing is based on the concept of spatial neural processing—the time synchrony between AP spikes arriving through different paths in a neural circuit^13^. This time-synchrony is influenced by the INS lengths along the circuit paths as they change the CV. Our results showed that despite the observed reduction of CV, the actual CD can remain constant within the limit of compensation. Since CD is the actual parameter determining the time synchrony between different paths in a neural circuit, the above-mentioned traditional view is valid only after the limit of compensation has been reached. More importantly, beyond the compensation limit, not only the time synchrony but also the representation of the actual neural information is changed due to low-pass filtering effect of an INS. This is a component that was previously ignored in developing the theory of neural information processing, particularly in the pathological conditions related to myelin loss. Thus, our theory shows that this component must be added in developing such a theory while adjustments in spatial neural processing concept should be made in light of CD remaining constant within the compensation limit.

### Effect of radial components

**Figure 7:**
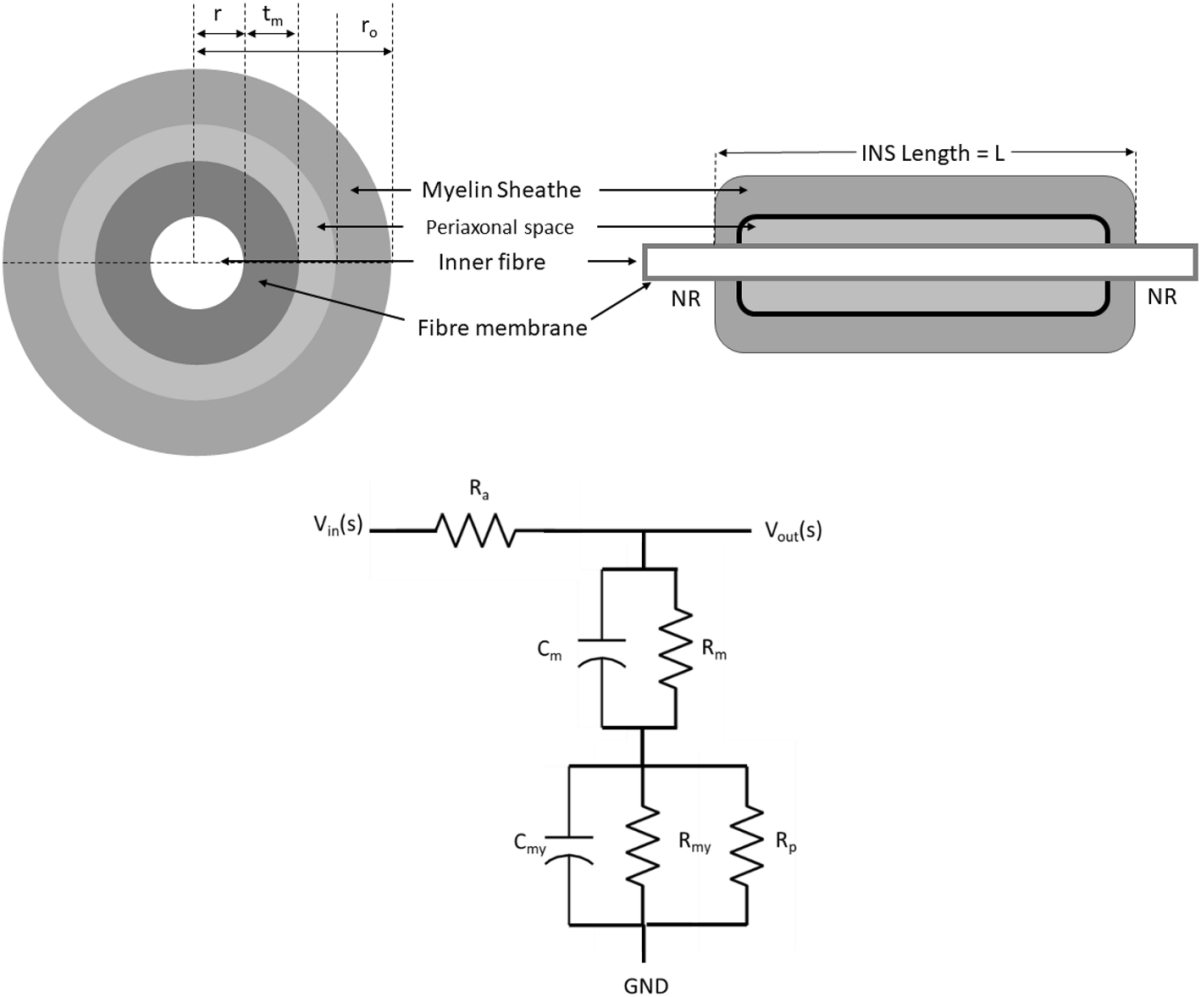
Myelin geometry and corresponding electrical circuit model used for modelling. Top figure shows the cross sectional and longitudinal view of myelin structure with its geometrical parameters. In the bottom, ${R}_{a}, {R}_{m}$, ${R}_{my}$ and ${R}_{p}$ are the resistances of the axoplasm, axolemma, myelin sheath and periaxonal space respectively and ${C}_{m}$ and ${C}_{my}$ are the capacitances of the axolemma and myelin sheath. Inputs and outputs have been shown in complex $s$-plane.

Our analyses were based on the DC model^18^ that didn’t explicitly include the effects of myelin radial components. Gow and Devaux^22^ showed that its absence decreases CV particularly in small fibres that are the largest constituents of CNS. Although initially thought that these radial components have adhesive properties to hold the myelin lamellae together, electrophysiological modelling in^23^ showed that they have insulative properties by formation of diffusion barriers and thereby regulating current flow through the myelin sheath. Using rigorous biophysical and ultrastructural techniques including X-ray diffraction,^24^ conclusively established this diffusion barrier property of the radial components. The main reason for not including it explicitly in our analyses is the lack of appropriate data. The effect of radial components on CV for small fibres described in^22^ used computational modelling techniques with two alternative models, one with the resistance resulting from the Tight Junction (TJ) of the radial component in series with myelin resistance–capacitance assembly; termed as the TJM, and the other with the resistance of TJ in parallel with myelin resistance in the conventional DC model—for ease of discussion we term this model as DCTJM. Electrical property of epithelial cell was used to characterise the electrical property of TJ. However, the works described in^19^,^21^ show that in CNS the electrical property of INS is strongly dependent on local Oligodendrocyte properties. Therefore, for CNS fibres, it seems more pragmatic to characterise the electrical properties of TJ in fibre-specific manner. Currently, to our knowledge, such data is unavailable for CC and CB fibres—the two small fibres explored in this work. Despite the lack of data, following the approach described in^22^, we have explored the possible implications of radial components on variations of ${f}_{L}$ in small fibres. The native configuration of the fibres is defined by their respective geometrical parameters given in Table 2, and thickness and resistivity of intramyelinic space as $d={t}_{m}$ and $\rho =1.96 \Omega m$^23^ respectively where ${t}_{m}$ is the thickness of a lipid layer (see Fig. 7, Section “Materials and methods). The main results could be summarised as follows:

1. From a system point of view, the TJM model proposed in^22^ introduced an extra zero in the TF of all fibres making a change in shape of their Bode magnitude plots as shown by the green curves in Fig. S7 (SI). For illustration purpose we have only depicted the response of the largest fibre $A\alpha 11$ and the two smallest fibres CC and CB. However, the location of that extra zero is far away from their corresponding ${f}_{L}$ and therefore practically has no effect in the system’s response up to their respective ${f}_{L}$. On the other hand, the number of zeros and poles of the DCTJM is the same as the DC model used here. Effectively, all the three models imply that an INS is a lowpass filter.
2. Consistent with the results of^22^ we found that for large fibres ($A\alpha 11$ here) there is no effect of TJ on ${f}_{L}$ (Fig. S8, SI) in its native configuration for all the three models. For small fibres (CB and CC) the TJM and the DC model used here gives no difference in ${f}_{L}$ (Fig. S9, SI). However, in the DCTJM the ${f}_{L}$ is reduced by 22.32 Hz for CB and by 24.67 Hz for CC compared to the other two models.
3. The above-mentioned phenomenon for CC and CB is strongly dependent on the value of $d$. For $d<{t}_{m}$, no difference between the three models could be found (Fig. S10, SI). On the other hand, for $d\ge {t}_{m}$, although the TJM and the DC model show the same characteristics, marked difference has been observed in the case of the DCTJM (Fig. S11, SI). In this case ${f}_{L}$ is reduced by 150.16 Hz for CB and by 186 Hz for CC.
4. The linear interdependency of $L$, $r$ and $M$, irrespective of the type of nerve fibres still holds true with the TJM and DCTJM model (Fig. S12, SI).

Effectively we found that even for small fibres, the TJM and the DC model used here always give the same results as far as ${f}_{L}$ is concerned but the DCTJM gives different results depending on the chosen thickness of intramyelinic space. Consistent with^18^, these results underscore the important role played by the periaxonal resistance ${R}_{p}$. In all the three models, ${R}_{p}$ is in parallel with their respective myelin-TJ assembly resistance and therefore the systems’ response is strongly dependent on their relative values. This is also reflected by the dependence of the system’s response on $d$ which determines the value of the resistance of TJ. Given the results in^19^,^21^ showing the region-specific difference in Oligodendrocyte properties, and hence the fibres’ electrical characteristics in CNS, these results show that fibre-specific characterization of ${R}_{p}$ and myelin-TJ assembly resistance (in essence, the value of $d$) is necessary to fully characterize the effects of radial components on frequency domain properties of INS in these fibres.

### Limitations of the proposed theory

The first limitation of our theory is that here we considered INS as an isolated system and “clean” turn-by-turn myelin loss. In practice, loss of myelin is “patchy”. This limitation could be overcome by partitioning an INS into a “normal” and a “patchy” region and then deriving TF for each region followed by multiplication of these TFs to create a composite TF. Subsequently, this TF could be used to obtain the results corresponding to those described in Section “Results”.

The second limitation is, although more than $1$ KHz AP firing rate has not been observed in practice, still our theory predicts that the NCC of a healthy fibre could be as high as 10 KHz. Given the observation in Fig. S6 (SI) which shows that according to the condition $L/M$ $\le 0.5 \mu$ m presented in Section "*L*, *r* and *M* are linearly interdependent irrespective of the type of nerve fibres", no PNS fibre could maintain ${f}_{L}\ge 1$ KHz for $M\le 3$, apparently this is to increase the resilience of a fiber against possible myelin loss. However, we would like to point out that this value is dependent on the choice of ${R}_{a}$, ${R}_{m}$, ${R}_{my}$, ${C}_{m}$ and ${C}_{my}$ (see Section “Materials and methods”) which in turn depend on the biological constants ${\rho }_{a}$, ${\rho }_{m}$, ${\epsilon }_{m}$ and ${t}_{m}$. As discussed in the Section “Materials and methods”, there are considerable variations in numerical values of these parameters reported over the years. Using their different values results in different numerical values of ${f}_{L}$. We are unable to comment on such variations of these physical constants measured in different experiments, but this wide range of variation certainly indicates that not only the ${f}_{L}$ but all the other quantitative results derived in Section “Results”, can only be considered accurate with respect to the numerical values we have used in Table 1—Section “Materials and methods”. However, even using different values of the above-mentioned constants the phenomena we described here remain the same. Therefore, we tried to avoid providing quantitative results wherever possible and preferred describing the essential parts of our theory in its functional form. We suggest that more experiments are needed to measure these values accurately to confirm the numerical values of the results we presented in Section “Results”.

**Table 1:** Electrical parameters used in this work.

Parameter name	Value	Reference
Axoplasm resistivity (ρ_a_)	2 Ωm	
Periaxonal resistivity (ρ_1_)	0.53 Ωm	Estimated from^18^
Paranode resistivity (ρ_2_)	5.5 Ωm	Estimated from^18^
Membrane resistivity (ρ_m_)	3.8 × 10^8^ Ωm	Estimated from^21^
Axoplasm dielectric constant	60	^25^
Membrane dielectric constant	11	^25^
Free space permittivity	8.854 × 10^–12^ F/m	

The third limitation is, the compensatory phenomenon predicted by our theory, although widely speculated by several authors, is yet to be detected experimentally. Therefore, we suggest directed experiments based on our theoretical arguments to either prove or disprove such phenomenon which will give more insights into the operation of our nervous system under pathological conditions.

## Materials and methods

### The modelling circuit structure and electrical parameters estimation

The structure of the INS we used for modelling purpose is shown in Fig. 7 along with the notations we used to specify its geometry.

We approach the modelling considering cylindrical symmetry at the granularity of number of myelin turns rather than the traditional approach of using outer diameter since we are more interested to understand how the system behaves during changes in myelin turns.

We used the Double Cable (DC) model^18^—which includes the effect of periaxonal space—as the main circuit modelling structure of an INS (Fig. 7). To estimate the electrical components, we assume that all the myelin lamellae are already compacted with each lamella composed of two tightly apposed lipid bilayer (axolemma membrane)—a standard approach for modelling an INS. Consequently, if each lipid bilayer has a thickness of ${t}_{m}$, then the thickness of each myelin lamella is $2{t}_{m}$, and its capacitance and resistances could be expressed in terms of the corresponding parameters of axolemma as follows:

7a $$\mathrm{Myelin\,resistance }\,{R}_{my}=2M{R}_{m}$$

7b $$\mathrm{Myelin\,capacitance }\, {C}_{my}=\frac{{C}_{m}}{2M}$$

where ${R}_{m}$ and ${C}_{m}$ represent the resistance and capacitance of the axolemma which, for INS length $L$ could be expressed as

7c $${R}_{m}=\frac{{\rho }_{m}}{2\pi L}\left[\mathrm{ln}(1+\frac{{t}_{m}}{r})\right]$$

7d $${C}_{m}=\frac{2\pi {\epsilon }_{m}L}{\mathrm{ln}(1+\frac{{t}_{m}}{r})}$$

where ${\rho }_{m}$ and ${\epsilon }_{m}$ are the resistivity and permittivity of the axolemma respectively. On the other hand, the axoplasmic resistance could be given by

7e $${R}_{a}=\frac{{\rho }_{a}L}{\pi {r}^{2}}$$

where ${\rho }_{a}$ is the resistivity of the axoplasm. Following the cylindrical symmetry, assuming that the thickness of the periaxonal and paranodal space being ${d}_{1}$ and ${d}_{2}$ respectively with their lengths being ${L}_{1}$ and ${L}_{2}$ respectively, the corresponding resistances (${R}_{pa}$ and ${R}_{pn}$ respectively) could be calculated as

7f $${R}_{pa}=\frac{{\rho }_{1}{L}_{1}}{\left[\pi {(r+{t}_{m}+{d}_{1})}^{2}-\pi {(r+{t}_{m})}^{2}\right]}$$

7g $${R}_{pn}=\frac{{\rho }_{2}{L}_{2}}{\left[\pi {(r+{t}_{m}+{d}_{2})}^{2}-\pi {(r+{t}_{m})}^{2}\right]}$$

where ${\rho }_{1}$ and ${\rho }_{2}$ are the resistivity of the periaxonal and paranodal fluid respectively. The total periaxonal resistance is the sum of the above two resistances and thus could be expressed as

7h $${R}_{p}={R}_{pa}+{R}_{pn}$$

The choice of the fundamental electrical constants used in this work are shown in Table 1. We observed that the experimentally reported values for these parameters are different in different published papers. A specific example is ${\rho }_{a}$ which has been reported in the range of 3.3–0.7 Ωm^25–27^ making it difficult to determine which one to use. Therefore, in such cases we have used the mean value of the range.

${\rho }_{m}$, ${\rho }_{1}$ and ${\rho }_{2}$ have been derived from the experimentally measured specific conductivity values reported in^18^,^20^] respectively using the following general conversion formula for a cylinder with length $L$, inner radius $r$ and thickness $d$

7i $$\rho =\frac{2\pi LR}{\mathrm{ln}[1+\frac{d}{r}]};\mathrm{ where}, R=\frac{1}{2\pi rL{g}_{m}}$$

${g}_{m}$ being the specific conductivity of the membrane of the cylinder in question.

### Geometrical parameters

Table 2 showed the geometrical dimensions of the fibers we use in this work. The types of PNS fibres we explored are: $A\alpha I$, $A\beta I$ and $A\delta I$, each with two types of geometry. Therefore, for clarity we denote them as $A\alpha 11$ and $A\alpha 12$, $A\beta 11$ and $A\beta 12$, and $A\delta 11$ and $A\delta 12$ in Table 2. The CNS fibres explored are from Cerebellum and Corpus Callosum taken from^18^, denoted as CB and CC respectively.

**Table 2:** Geometrical data of the fibers under consideration.

Axon type	Inner radius ($r$) µm	Outer radius ($ro$) µm	No. of myelin turns ($M$)	INS length ($L$) µm	$g$-ratio ($r/ ro$)	$\gamma = ro/L$	References
$A\alpha 11$	10	14	400	2000	0.7142	0.007	^28^
$A\alpha 12$	6.5	9.1	260	1300	0.7142	0.007	^28^
$A\beta 11$	6	8.4	240	1200	0.7142	0.007	^28^
$A\beta 12$	3	4.2	120	600	0.7142	0.007	^28^
$A\delta 11$	2.5	3.5	100	500	0.7142	0.007	^28^
$A\delta 12$	0.5	0.7	20	100	0.7142	0.007	^28^
$CC$	0.18	0.25	7	79.1	0.72	0.003	^21^
$CB$	0.36	0.49	13	106	0.734	0.0046	^21^

Given the inner and outer radius of the fibers, the number of myelin turns ($M$), $g$-ratio and $\gamma$ (a parameters we use here which is quantitatively the inverse of $L/D$ in^1^) have been calculated using the following equations

7j $$M= \frac{{r}_{o}-r}{{2t}_{m}}$$

7k $$g=\frac{r}{{r}_{o}}=\frac{1}{1+\frac{2M{t}_{m}}{r}}$$

7l $$\gamma =\frac{{r}_{o}}{L}=\frac{1+\frac{2M{t}_{m}}{r}}{L/r}$$

The radius of the periaxonal space (${d}_{1}$) and paranode (${d}_{2}$) has been considered as 12 nm and 7 nm respectively according to^20^ whilst the cell membrane thickness = 5 nm used for all the fibers have been estimated from^18^. We also consider that the length of periaxonal space (${L}_{1}$) and the corresponding paranode (${L}_{2}$) are 90% and 10% of the length of INS respectively in the absence of corresponding data.

### INS system modelling and${f}_{L}$ calculation

By nature, the circuit shown in Fig. 7 is a Linear Time Invariant (LTI) system and therefore $s$-plane Transfer Function (TF) based system analysis approach is applicable for its characterization. In such an approach, the locations of the complex poles and zeros of the system TF describe the system’s behaviour completely in terms of its Gain (amplification/attenuation of the input signal at its output), frequency and temporal response. Referring to Fig. 7, the TF could be derived as follows.

The total radial impedance of the axolemma can be given by

8 $$\frac{1}{{Z}_{m}}=\frac{1}{{R}_{m}}+s{c}_{m}; \,{Z}_{m}=\frac{{R}_{m}}{1+s{R}_{m}{c}_{m}}$$

Similarly, the total radial impedance of myelin including the resistance of periaxonal space is given by

9 $$\frac{1}{{Z}_{my}}=\frac{1}{{R}_{my}}+\frac{1}{{R}_{p}}+s{c}_{m};\,{Z}_{my}=\frac{{R}_{my}{R}_{p}}{{R}_{p}+{R}_{my}+s{R}_{my}{R}_{p}{c}_{my}}$$

The total effective radial impedance is thus given by

10 $${Z}_{eff}={Z}_{my}+{Z}_{m}=\frac{{R}_{m}}{1+s{R}_{m}{c}_{m}}+\frac{{R}_{my}{R}_{p}}{{R}_{p}+{R}_{my}+s{R}_{my}{R}_{p}{c}_{my}}$$

Using the potential divider rule, the output voltage of the circuit in Fig. 7 could be given by

11 $${v}_{0}\left(s\right)=\frac{{Z}_{eff}}{{R}_{a}+{Z}_{eff}}{v}_{in}(s)$$

Therefore, the generalized form of TF of an INS could be given as,

12 $$H\left(s\right)=\frac{{V}_{o}\left(s\right)}{{V}_{in}\left(s\right)}=\frac{{Z}_{eff}}{{R}_{a}+{Z}_{eff}}=\frac{{R}_{m}{R}_{eqv}\left({C}_{m}+{C}_{my}\right) s+\left({R}_{m}+{R}_{eqv}\right)}{{C}_{m}{C}_{my}{R}_{a}{R}_{m}{ s}^{2}+s\left({C}_{m}{R}_{a}{R}_{my}+{C}_{my}{R}_{a}{R}_{eqv}+{C}_{my}{R}_{m}{R}_{eqv}\right)+\left({R}_{a}+{R}_{m}+{R}_{eqv}\right)}=\frac{as+b}{c{s}^{2}+ds+e}$$

where ${R}_{eqv}=\frac{{R}_{my}{R}_{p}}{{R}_{my}+{R}_{p}}$. Subsequently, the frequency-dependent gain of the system could be given by $G\left(\omega \right)=\left|H(j\omega )\right|$ with $s=j\omega$, $\omega$ being the angular frequency. One may extract ${f}_{L}$ from $G\left(\omega \right)$ as follows: considering the membrane resting potential and the membrane potential at which Na channel activates are $- 70$ mV and $- 55$ mV respectively, with the peak of an AP being $+ 40$ mV, to trigger an AP at an NR its membrane potential must be at least $+ 15$ mV at the peak of an AP. This leads to the following condition for triggering an AP at the NR:

13 $$G(\omega )\ge 20\,log\frac{15}{40}= -8.519\,dB$$

Accordingly, ${\omega }_{L}=2\pi {f}_{L}$ represents the frequency at which $G\left({\omega }_{L}\right)=-8.519 dB$, where ${\omega }_{L}$ and ${f}_{L}$ are the angular and linear frequency respectively. This could be detected by examining the Bode magnitude plots which describes the characteristics of $G(\omega )$ vs $\omega$ of a TF.

### The simulation flow

Our main simulation experimental process could be described as follows—first we calculate the TF for each of the fibers using their geometrical and electrical parameters given in Tables 2 and 1 respectively and then replacing them in the circuit structure shown in Fig. 7. Subsequently, we calculated $G(\omega )$ for each of the fibres using Bode Magnitude plot and extracted the corresponding ${f}_{L}$ following Eq. (13). This step gives the system behaviour of an INS of that fibre in its normal operating conditions. Next, we progressively reduced $M$ for each fibre from their values given in Table 2 down to 1, which represents the limit of myelin loss to simulate the scenario of changes in myelin turns. At each step of this process, we repeat the process of calculating TF and corresponding ${f}_{L}$ that shows how ${f}_{L}$ changes with respect to the changes in myelin turns. Throughout the process the original $L$ and $r$ of a fibre (given in Table 2) has been kept constant. The data thus generated has been used for deriving the rest of our results.

### Calculation of CD and CV

In system theory, a phase delay is the measure of how a certain frequency component of a signal is delayed by the system and therefore is more applicable for analysis of pure sinusoidal signals. On the other hand, the group delay gives the measure of the delay introduced by the system to the overall envelope of a signal composed of multiple frequency components. Since an AP is not a pure sinusoid but a composite signal, the time delay of its conduction through an INS must be estimated as a group Conduction Delay (CD) according to Eq. (14) given below:

14 $$CD=- \frac{d\theta (\omega )}{d\omega }$$

where $\theta (\omega )$ is the frequency-dependent phase change introduced to an input signal by the system which could be directly calculated from the Bode Phase plot of the system TF. Therefore, for each TF created using the simulation flow mentioned above, we derived the CD directly from the corresponding Bode Phase plot by applying Eq. (10). Consequently, for an INS length $L$, CV could be calculated as $CV=L/CD$.

### Supplementary Information

Supplementary Figures.

## Data Availability

We have used publicly available data as referenced in Section “Materials and methods”. The modelling codes are available at: https://www.dropbox.com/scl/fo/95qgx45nuvcyjet5ej6ra/h?rlkey=l1wjq57li37wd2qcuj6jgbndp&dl=0.

## References

[1] Rushton WAH (1951). A theory of the effects of fibre size in medullated nerve. J. Physiol..

[2] Waxman S, Bennett M (1972). Relative conduction velocities of small myelinated and non-myelinated fibres in the central nervous system. Nat. New Biol..

[3] Smith R, Koles Z (1970). Myelinated nerve fibers: Computed effect of myelin thickness on conduction velocity. Am. J. Physiol..

[4] Stassart RM, Möbius W, Nave K-A, Edgar JM (2018). The axon-myelin unit in development and degenerative disease. Front. Neurosci..

[5] Villalón E (2018). Internode length is reduced during myelination and remyelination by neurofilament medium phosphorylation in motor axons. Exp. Neurol..

[6] Beuche W, Friede RJ (1985). A new approach toward analyzing peripheral nerve fiber populations. II. Foreshortening of regenerated internodes corresponds to reduced sheath thickness. J. Neuropathol. Exp. Neurol..

[7] Gattuso JM, Glasby MA, Gschmeissner SE (1988). Recovery of peripheral nerves after surgical repair with treated muscle grafts. Neuro-Orthopedics.

[8] Cragg BG, Thomas PK (1964). The conduction velocity of regenerated peripheral nerve fibres. J. Physiol..

[9] Hildebrand C, Kocsis JD, Waxman GS (1985). Myelin sheath remodeling in regenerated rat sciatic nerve. Brain Res..

[10] Tomassy GS (2014). Distinct profiles of myelin distribution along single axons of pyramidal neurons in the neocortex. Science.

[11] Ford MC (2015). Tuning of Ranvier node and internode properties in myelinated axons to adjust action potential timing. Nat. Commun..

[12] Suminaite D, Lyons DA, Livesey MR (2019). Myelinated axon physiology and regulation of neural circuit function. Glia.

[13] Bonetto G, Belin D, Káradóttir RT (2021). Myelin: A gatekeeper of activity-dependent circuit plasticity?. Science.

[14] Williamson JM, Lyons DA (2018). Myelin dynamics throughout life: An ever-changing landscape?. Front. Cell. Neurosci..

[15] Kostal L, Lansky P, Rospars J-P (2007). Neuronal coding and spiking randomness. Eur. J. Neurosci..

[16] Panzeri S, Macke JH, Gross J, Kayser C (2015). Neural population coding: Combining insights from microscopic and mass signals. Trends Cognit. Sci..

[17] Burgess P, Perl E, Iggo A (1973). Cutaneous mechanoreceptors and nociceptors. Handbook of sensory physiology.

[18] Cohen CC, Popovic MA, Klooster J, Weil M, Moebius W, Nave K, Kole MHP (2020). Saltatory conduction along myelinated axons involves a periaxonal nanocircuit. Cell.

[19] Call CL, Bergles DE (2021). Cortical neurons exhibit diverse myelination patterns that scale between mouse brain regions and regenerate after demyelination. Nat. Commun..

[20] Chomiak T, Hu B (2009). What is the optimal value of the g-ratio for myelinated fibers in the rat CNS? A theoretical approach. PLoS ONE.

[21] Bakiri Y, Káradóttir RK, Cossell L, Attwell D (2011). Morphological and electrical properties of oligodendrocytes in the white matter of the corpus callosum and cerebellum. J. Physiol..

[22] Gow A, Devaux J (2008). A model of tight junction function in CNS myelinated axons. Neuron Glia Biol..

[23] Devaux J, Gow A (2008). Tight junctions potentiate the insulative properties of small CNS myelinated axons. J. Cell Biol..

[24] Denninger AR (2015). Claudin-11 tight junctions in myelin are a barrier to diffusion and lack strong adhesive properties. Biophys. J..

[25] Brosseau C, Sabri E (2021). Resistor–capacitor modeling of the cell membrane: A multiphysics analysis. J. Appl. Phys..

[26] Cole KS (1975). Resistivity of axoplasm. I. Resistivity of extruded squid axoplasm. J. Gen. Physiol..

[27] Barrett JN, Crill WE (1974). Specific membrane properties of cat motoneurones. J. Physiol..

[28] Kandel E, Schwartz J, Jessell TM (2000). Principles of Neural Science.

