# Supplementary material for: Filtering property of myelinated internode can change neural information representability and might trigger a compensatory action during demyelination
Source: Sci Rep. 2023 Dec 14;13:22227. doi: 10.1038/s41598-023-49208-9 (PMC10721845; doi:10.1038/s41598-023-49208-9)
Supplement: Supplementary file 1 — Supplementary Figures. [file 41598_2023_49208_MOESM1_ESM.docx]

**Supplementary Information**

Filtering property of myelinated internode can change neural information representability and might trigger a compensatory action during demyelination

Sarbani Das and Koushik Maharatna

School of Electronics and Computer Science, University of Southampton, University Road, Southampton, SO17 1BJ, United Kingdom

Corresponding author: Koushik Maharatna

**Email:**  [km3@ecs.soton.ac.uk](mailto:km3@ecs.soton.ac.uk)























Fig. S1. Bode plot for the fibers (from the top to bottom) $\boldsymbol{A\alpha}\boldsymbol{12, A\beta}\boldsymbol{11, A\beta}\boldsymbol{12, A\delta}\boldsymbol{11, A\delta}\boldsymbol{12}$, CC and CB. Solid circles denote $\boldsymbol{f}_{\boldsymbol{L}}$ for corresponding to the different myelin turns. All the fibres show the same characteristics – as $\boldsymbol{M}$ reduces, $\boldsymbol{f}_{\boldsymbol{L}}$ moves toward the lower frequency bands and vice-versa.


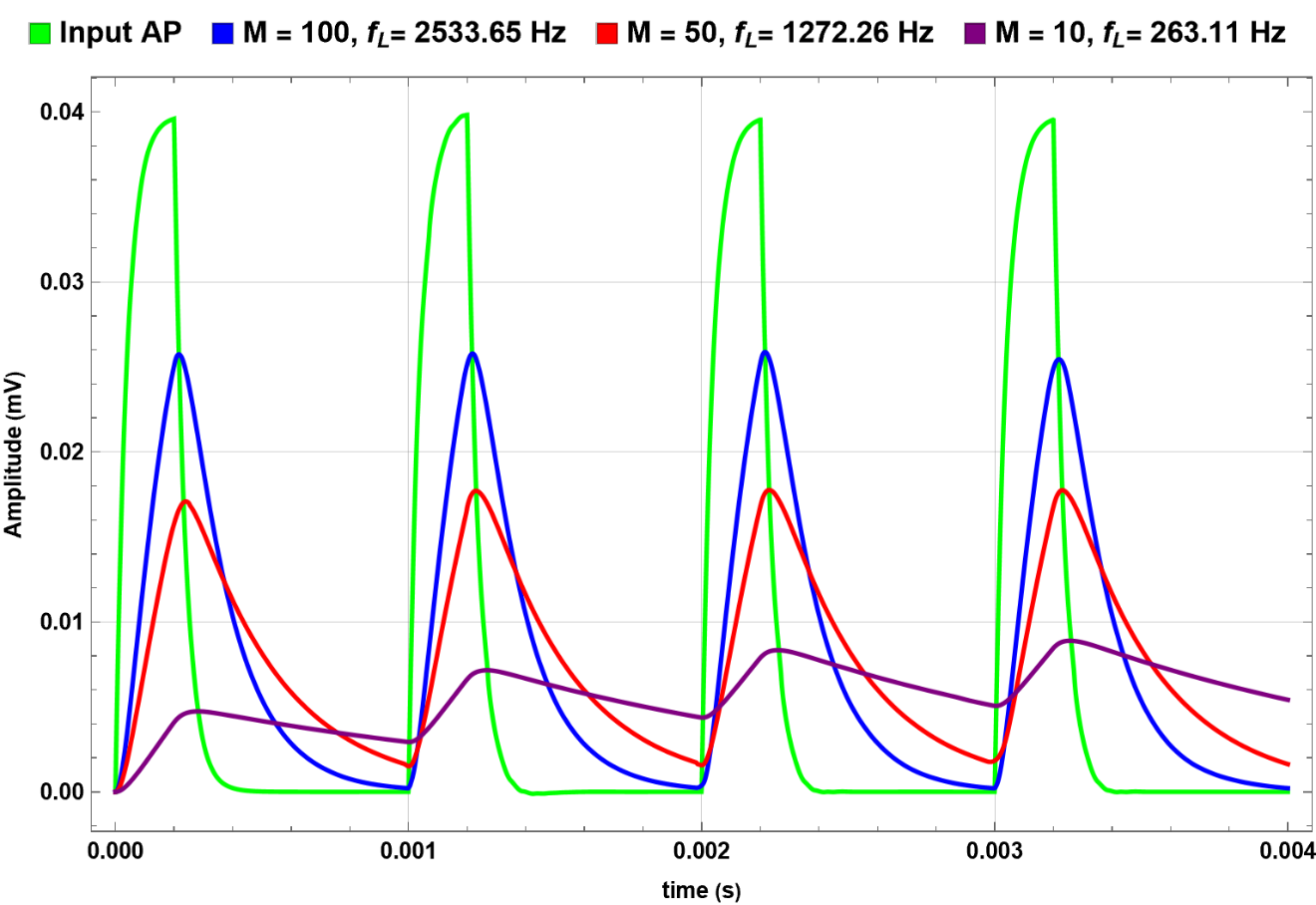


Fig. S2. Effect of lowpass filtering of INS for $\boldsymbol{A\alpha}\boldsymbol{11}$ fibre with different myelin turns: Synthetic AP train like those generated by integrate-and-fire model was created by passing a 1 KHz square wave with 20% duty cycle through a RC lowpass filter. For $\boldsymbol{M=10}\boldsymbol{0}$ and $\boldsymbol{M=5}\boldsymbol{0}$, the respective $\boldsymbol{f}_{\boldsymbol{L}}$ is higher than the input AP train frequency and consequently the corresponding output voltage of INS is greater than 15 mV, which is sufficient to generate an AP at the next NR (see subsection 4.3, Materials and Methods). But for $\boldsymbol{M=10}$, no AP can be generated at the next NR since the frequency of the input AP train is higher than the corresponding $\boldsymbol{f}_{\boldsymbol{L}}$ and thus, the output voltage of the INS is less than $\boldsymbol{15}$ mV. Consequently, for $\boldsymbol{M=10}$, the neural signal is disrupted.





Fig. S3. The loci of $\boldsymbol{f}_{\boldsymbol{L}}$ with change of myelin turns for the fibers considered in this work. The red dotted line represents $\boldsymbol{f}_{\boldsymbol{L}}\boldsymbol{=1}$ KHz limit. The rate of change of $\boldsymbol{f}_{\boldsymbol{L}}$ appears to be much steeper for the fibres with smaller $\boldsymbol{r}$ and $\boldsymbol{L}$ indicating dependence on these parameters too.








Fig. S4. Spectral property of an AP. Top figure shows AP generated using the classical Hodgkin-Huxley model and bottom figure shows the Power Spectral Density (PSD) of the AP. PSD was calculated by first taking Fourier Transform of the AP and then squaring the amplitudes of the individual Fourier components.








Fig. S5. The compensatory scenario for $\boldsymbol{A\alpha}\boldsymbol{11}$ fibre – top and bottom figures show the trend of reduction of $\boldsymbol{L}$ and $\boldsymbol{r}$ with reduction of $\boldsymbol{M}$ required to maintain $\boldsymbol{f}_{\boldsymbol{L}}\boldsymbol{=10}$ KHz. Note the difference of scales between their trends.





Fig. S6. The limiting condition for compensation in PNS fibres. Below $\boldsymbol{M}_{\boldsymbol{l}}\boldsymbol{=30}$ no more compensation is possible to maintain $\boldsymbol{f}_{\boldsymbol{L}}\boldsymbol{=10}$ KHz for any of the PNS fibres – note that for all the PNS fibres $\boldsymbol{M}_{\boldsymbol{l}}$ is overlapped. On the other hand, all PNS fibres can do compensation for $\boldsymbol{f}_{\boldsymbol{L}}\boldsymbol{=1}$ KHz (shown as the dotted line) until $\boldsymbol{M}_{\boldsymbol{l}}\boldsymbol{=3}$.


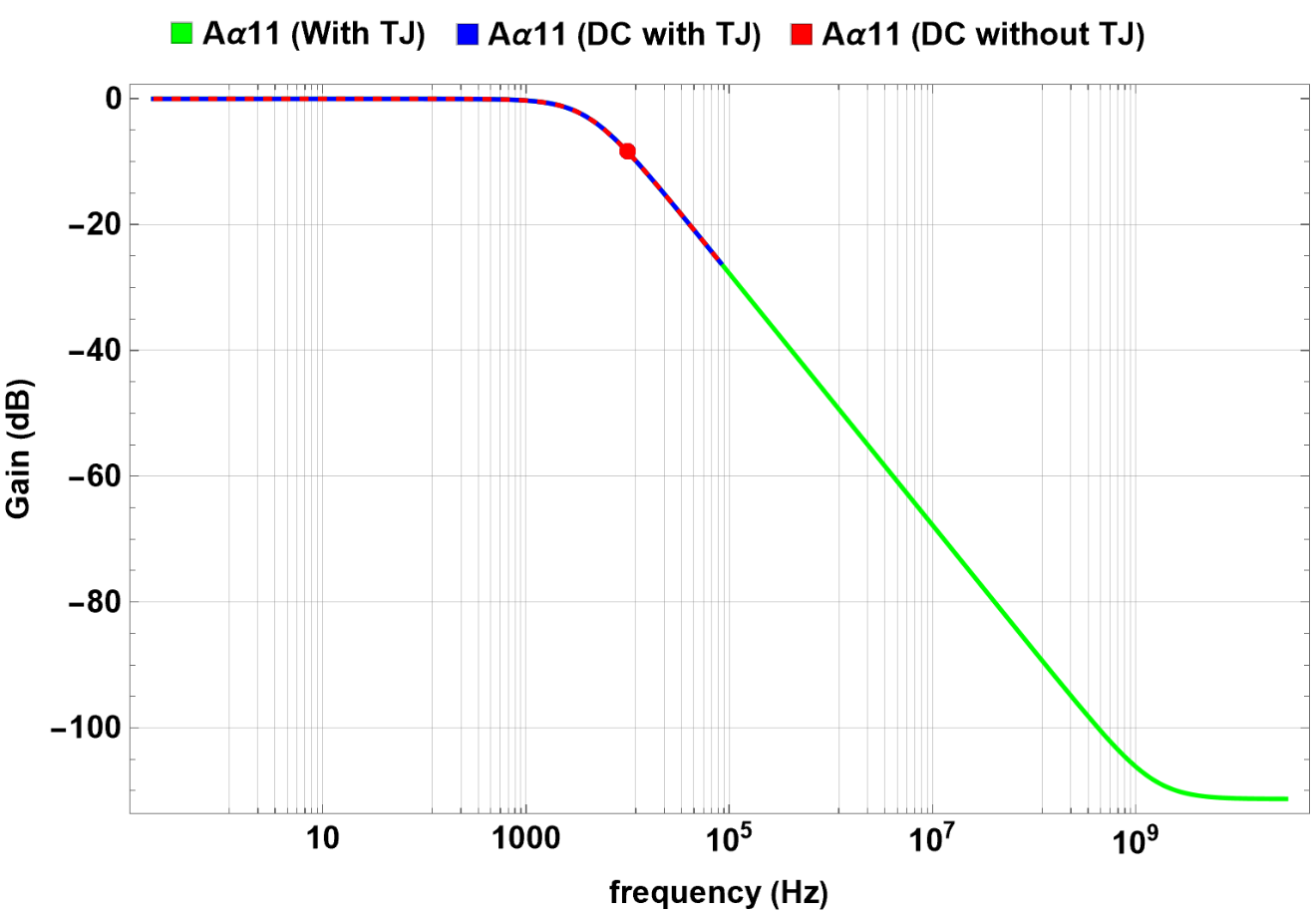


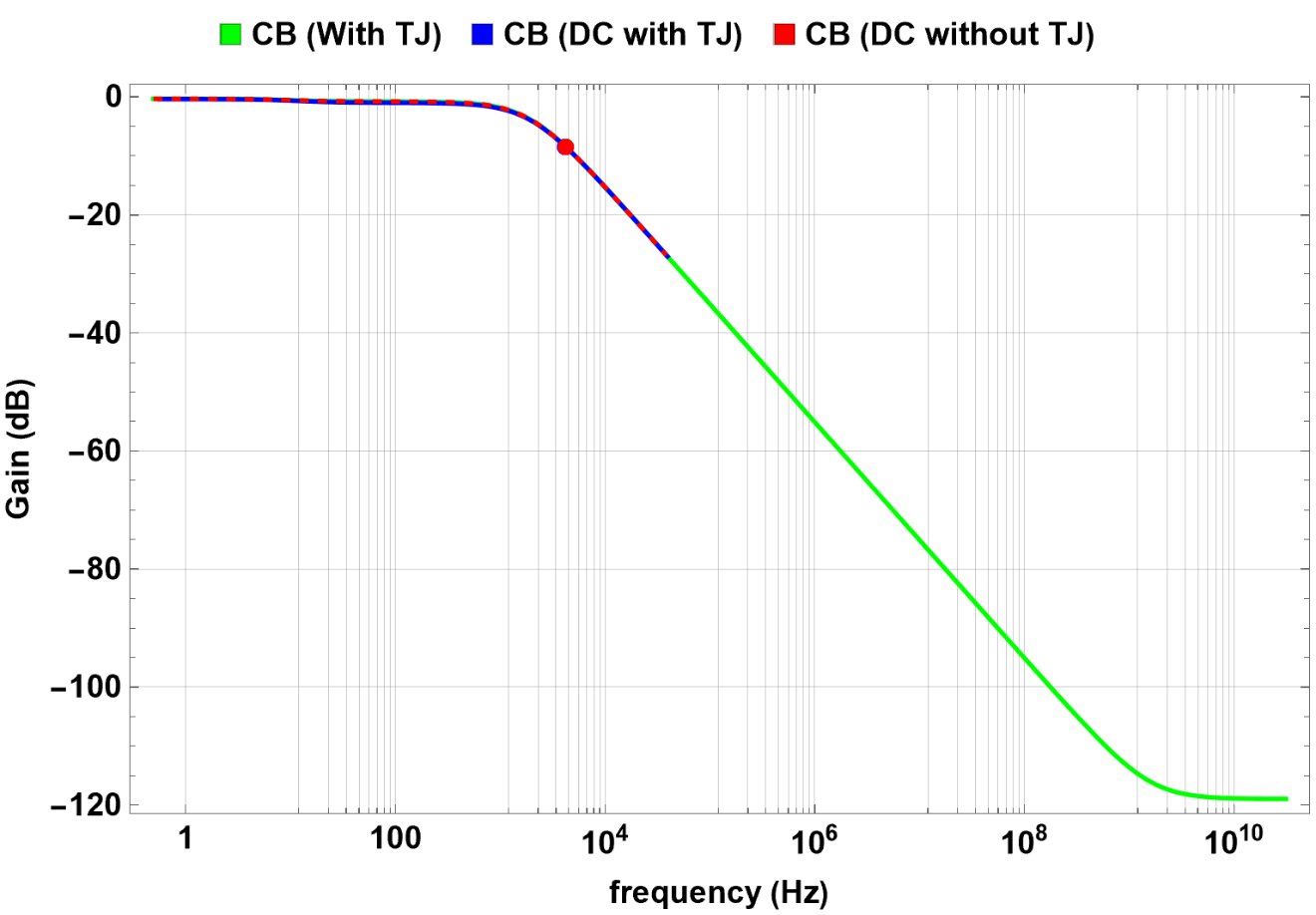


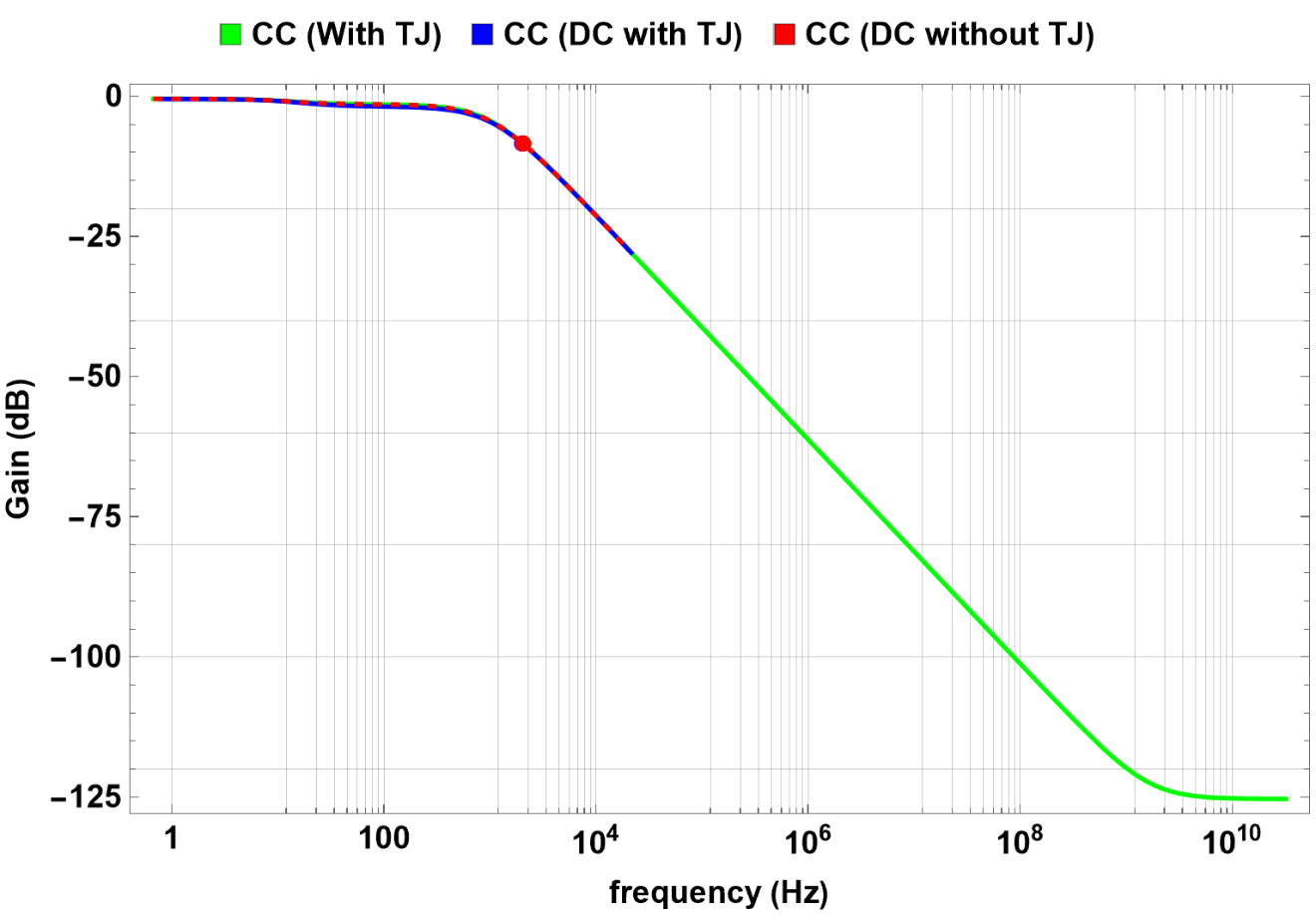


**Fig. S7**. Bode plot for the fibres in their native configuration following the TJM, DCTJM, and the DC model used here: (a) $A\alpha11$, (b) CB and (c) CC. Solid circles denote $f_{L}$ in each case. Thickness of intramyelinic space $d$has been considered equal to $t_{m}$ and resistivity of the intramyelinic space $\rho=1.96 Ωm$ was taken from [Josephson, R. K. & Schwab, W. E., 1979. *J Gen Physiol.*, 74(2), pp. 213–236]. For $A\alpha11$, there is no difference between the three models. For CC and CB, the characteristics of the TJM and the DC model used here are the same whereas the DCTJM shows a slight difference.





Fig. S8. Variation of $\boldsymbol{f}_{\boldsymbol{L}}$ with respect to $\boldsymbol{M}$ for $\boldsymbol{A\alpha}\boldsymbol{11}$ in all the three models in its native configuration. No difference has been observed between the models’ outputs. Solid circles denote the $\boldsymbol{f}_{\boldsymbol{L}}$ corresponding to each value of $\boldsymbol{M}$





(a)


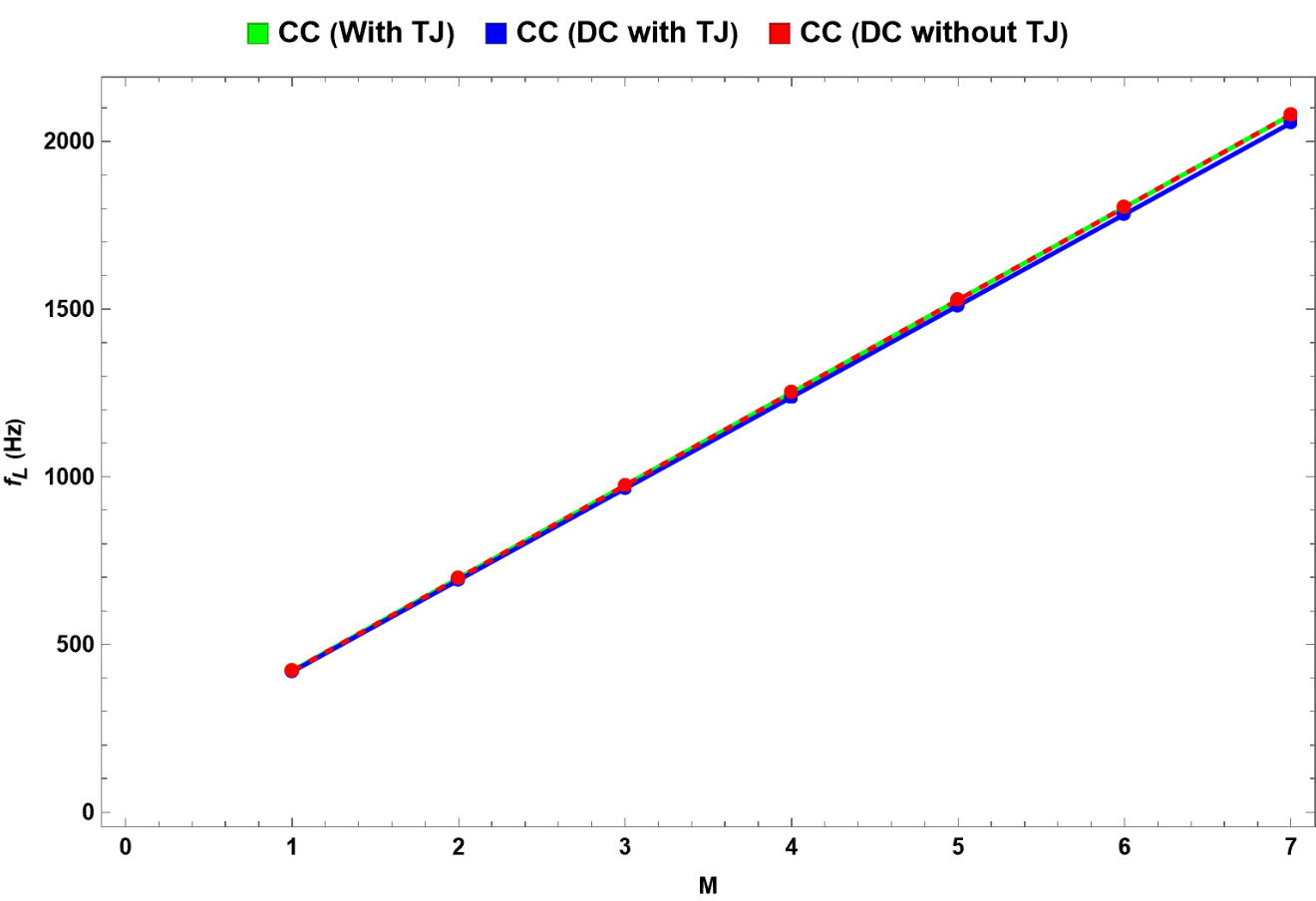


(b)

Fig. S9. Variation of $\boldsymbol{f}_{\boldsymbol{L}}$ for small fibres with respect to $\boldsymbol{M}$ in their native configuration: (a) CB and (b) CC. For both fibres, the TJM and the DC model used here show identical results whereas the DCTJM shows slight difference. Solid circles denote the $\boldsymbol{f}_{\boldsymbol{L}}$ corresponding to each $\boldsymbol{M}$


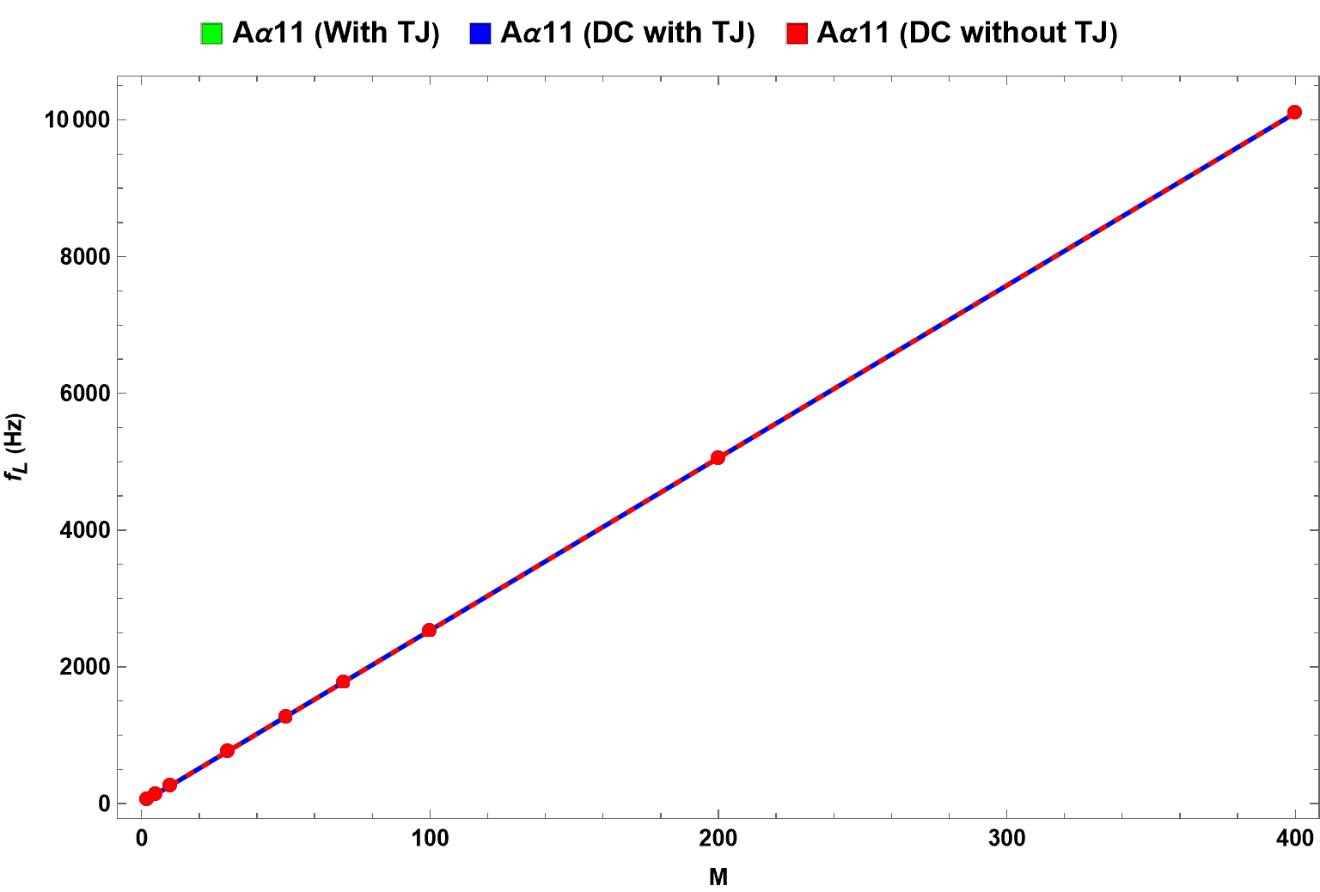


(a)


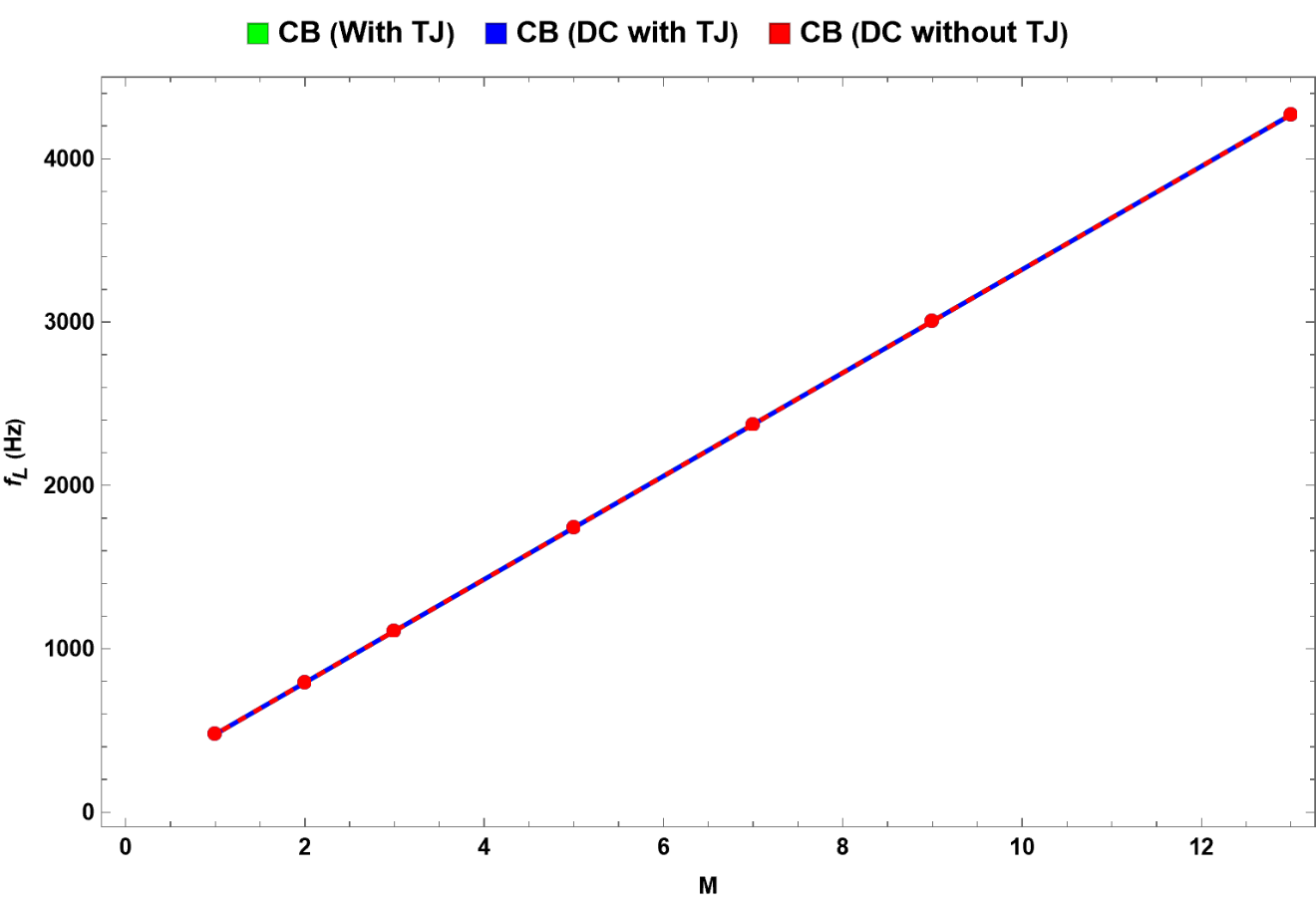


(b)


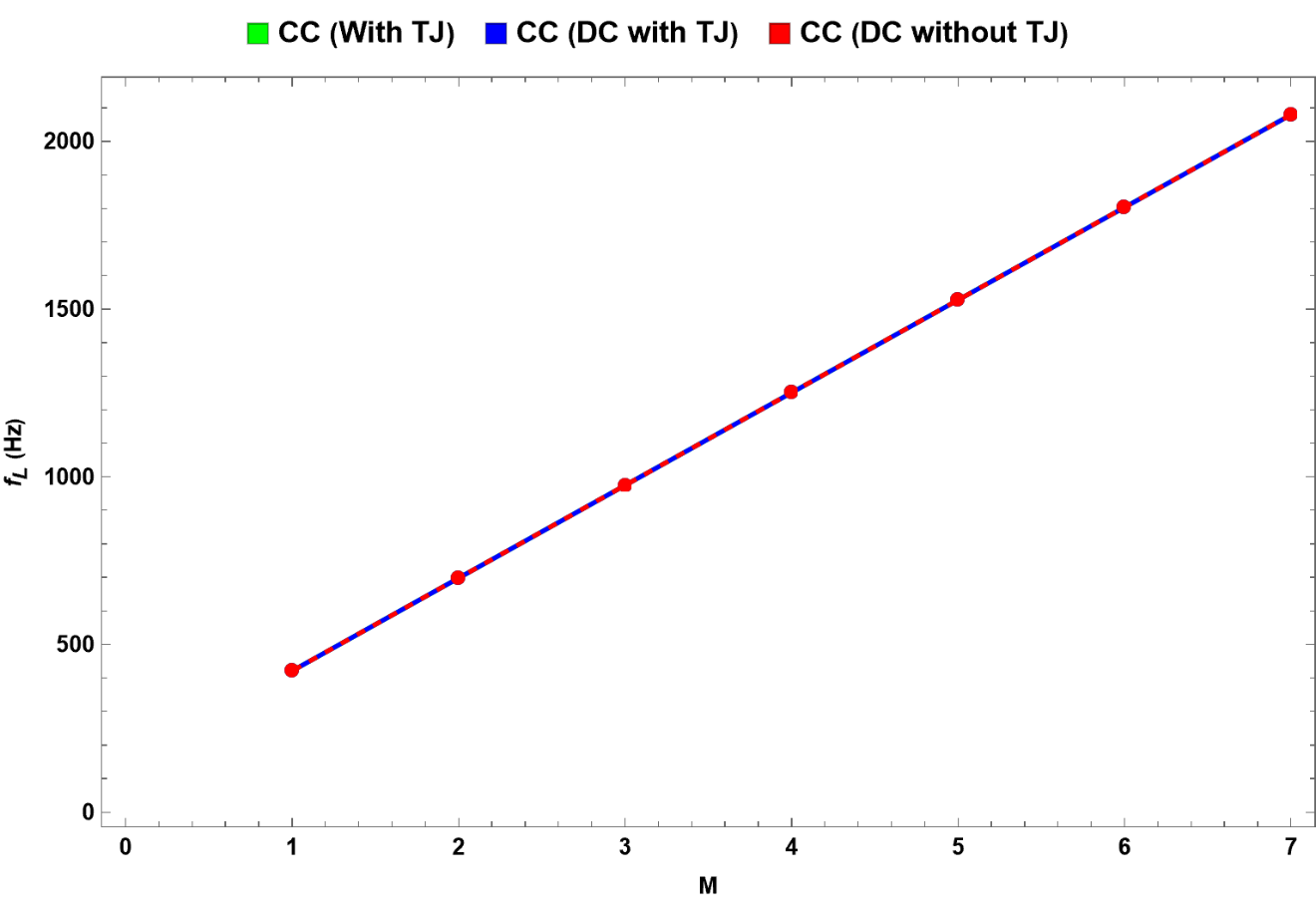


(c)

Fig. S10. Variation of $\boldsymbol{f}_{\boldsymbol{L}}$ for all fibres with respect to $\boldsymbol{M}$ when $\boldsymbol{d=0.01}\boldsymbol{t}_{\boldsymbol{m}}$: (a) $\boldsymbol{A\alpha}\boldsymbol{11}$, (b) CB, (c) CC. No difference between the models could be found.


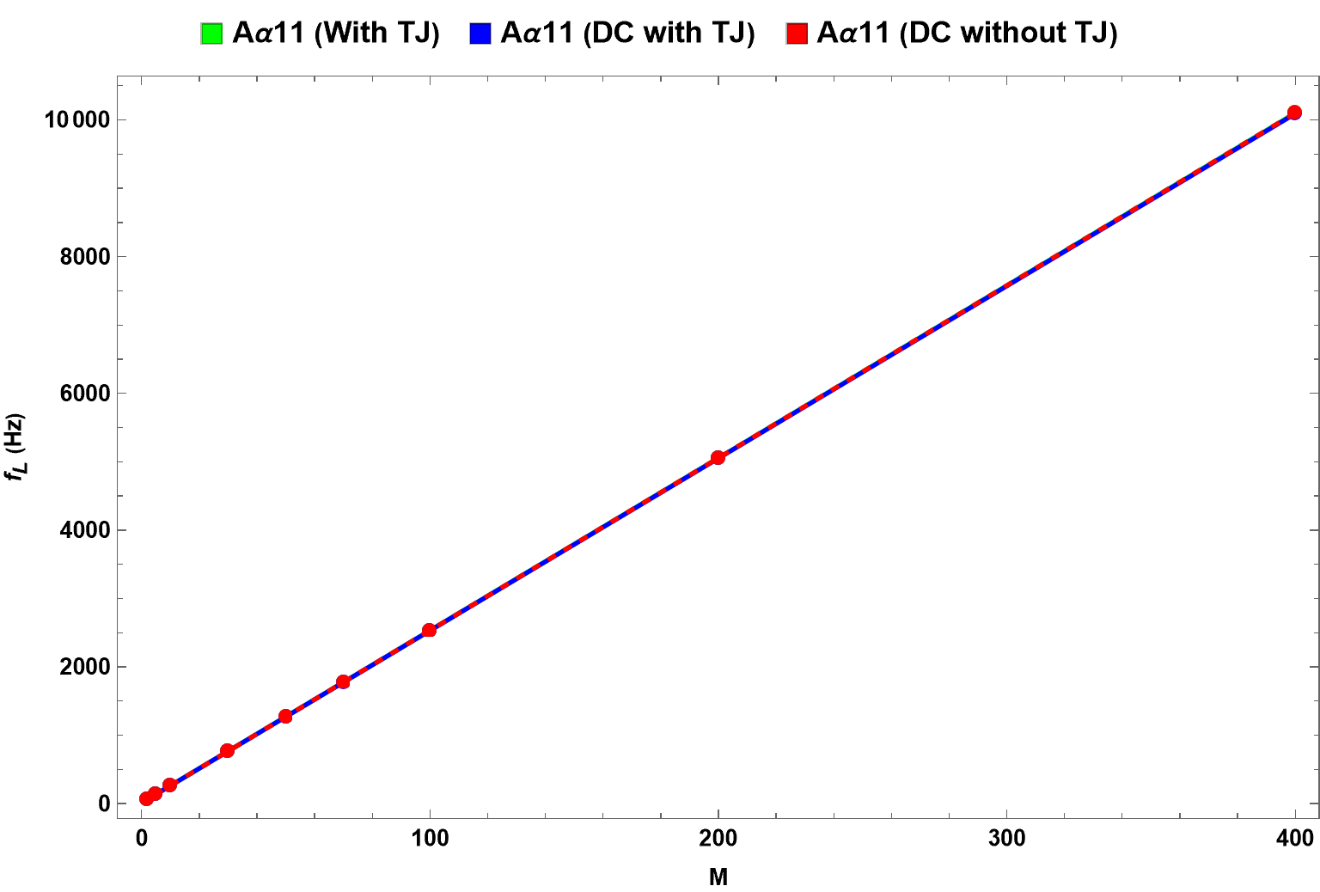


(a)


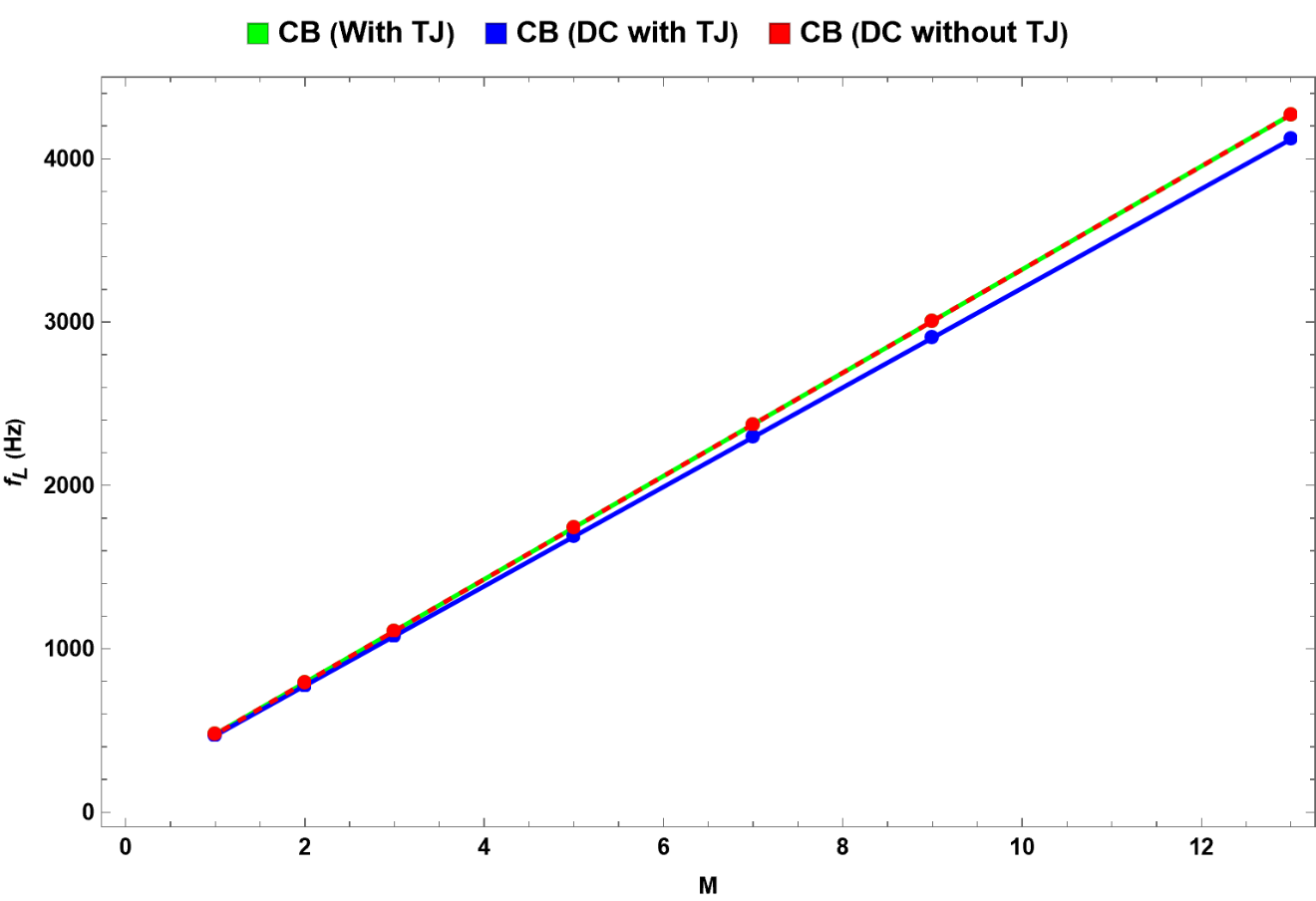


(b)


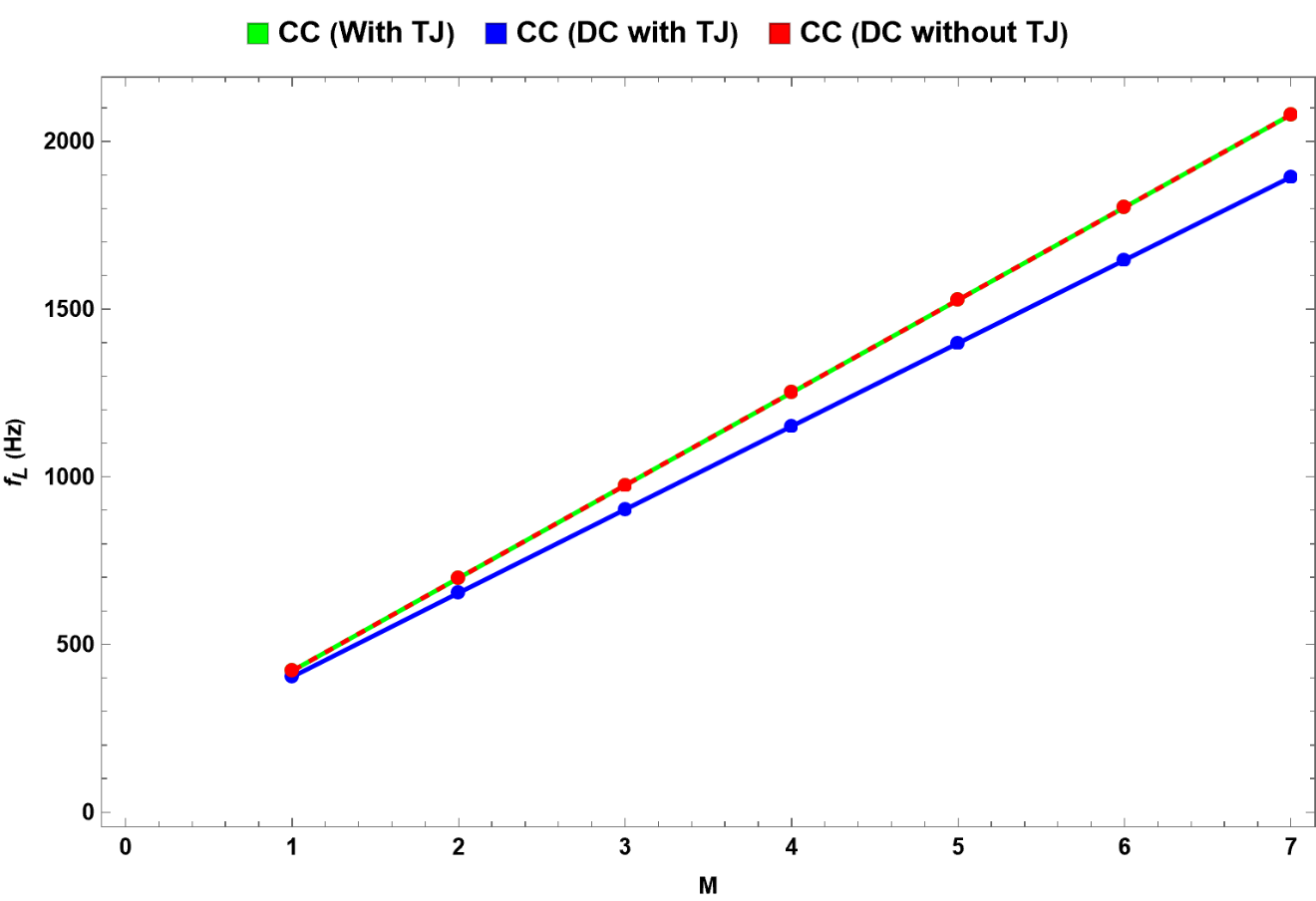


(c)

**Fig. S11**. Variation of $f_{L}$ for all the fibres with respect to $\boldsymbol{M}$ with $d=30 nm (6\times t_{m})$: (a) $A\alpha11$, (b) CB, and (c) CC. The results show that the effect of $d\geq t_{m}$ is minimal for large fibre $A\alpha11$. But it significantly alters the characteristics of the small fibres CB and CC. Once again, the TJM and the DC model used here give identical results, but the DCTJM gives significantly different results.


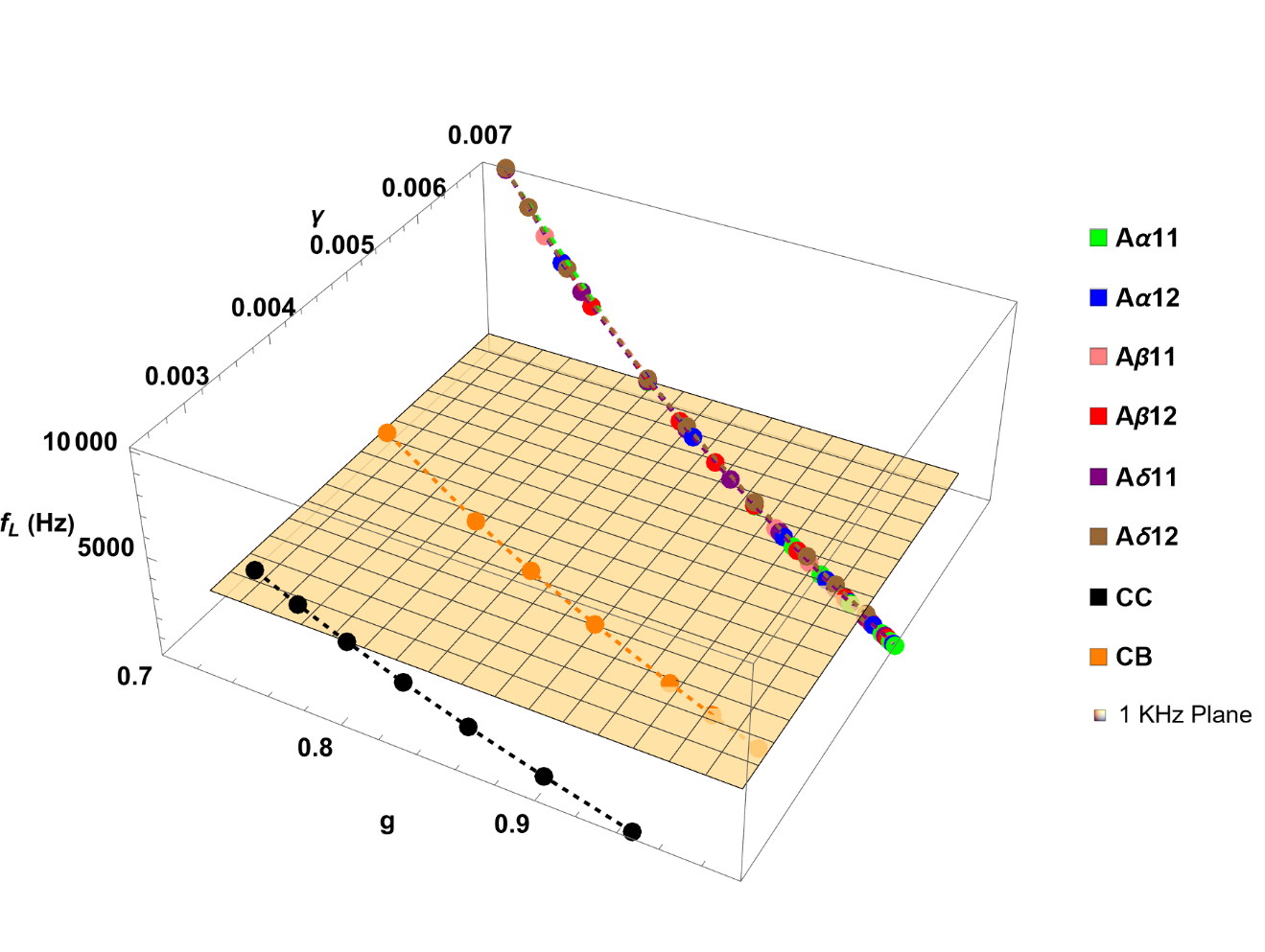


(a)





(b)

**Fig. S12**. Variation of $f_{L}$ for all the fibres with respect to $g$ and $\gamma$ for: (a) TJM, and (b) DCTJM. Linear interdependence between $f_{L}$, $g$ and $\gamma$ holds true in both models. The loci of $f_{L}$ for PNS fibres could be represented as the fitted linear curve $-23012.26-1264.82g+4.86\times{10}^{6}\gamma(R^{2}=0.99)$ and $-23009.73-1247.66g+4.85\times{10}^{6}\gamma(R^{2}=0.99)$ respectively with 99% confidence interval.
